# Correction: Phase separation of *Arabidopsis *EMB1579 controls transcription, mRNA splicing, and development

**DOI:** 10.1371/journal.pbio.3002095

**Published:** 2023-04-06

**Authors:** Yiling Zhang, Zhankun Li, Naizhi Chen, Yao Huang, Shanjin Huang

The Data Availability statement is incomplete. The complete statement should read:

RNA-seq and ChIP-seq data have been deposited at BioProject (https://www.ncbi.nlm.nih.gov/bioproject/) under the accession numbers PRJNA653772 and PRJNA945849, respectively. All other relevant data are in the manuscript and Supporting Information files.

## References

[1] Zhang Y, Li Z, Chen N, Huang Y, Huang S (2020) Phase separation of Arabidopsis EMB1579 controls transcription, mRNA splicing, and development. PLoS Biol 18(7): e3000782. 10.1371/journal.pbio.3000782 32692742 PMC7413564

