# Supplementary figures and images for: Phase separation of Arabidopsis EMB1579 controls transcription, mRNA splicing, and development
Source: PLoS Biol. 2020 Jul 21;18(7):e3000782. doi: 10.1371/journal.pbio.3000782 (PMC7413564; doi:10.1371/journal.pbio.3000782)

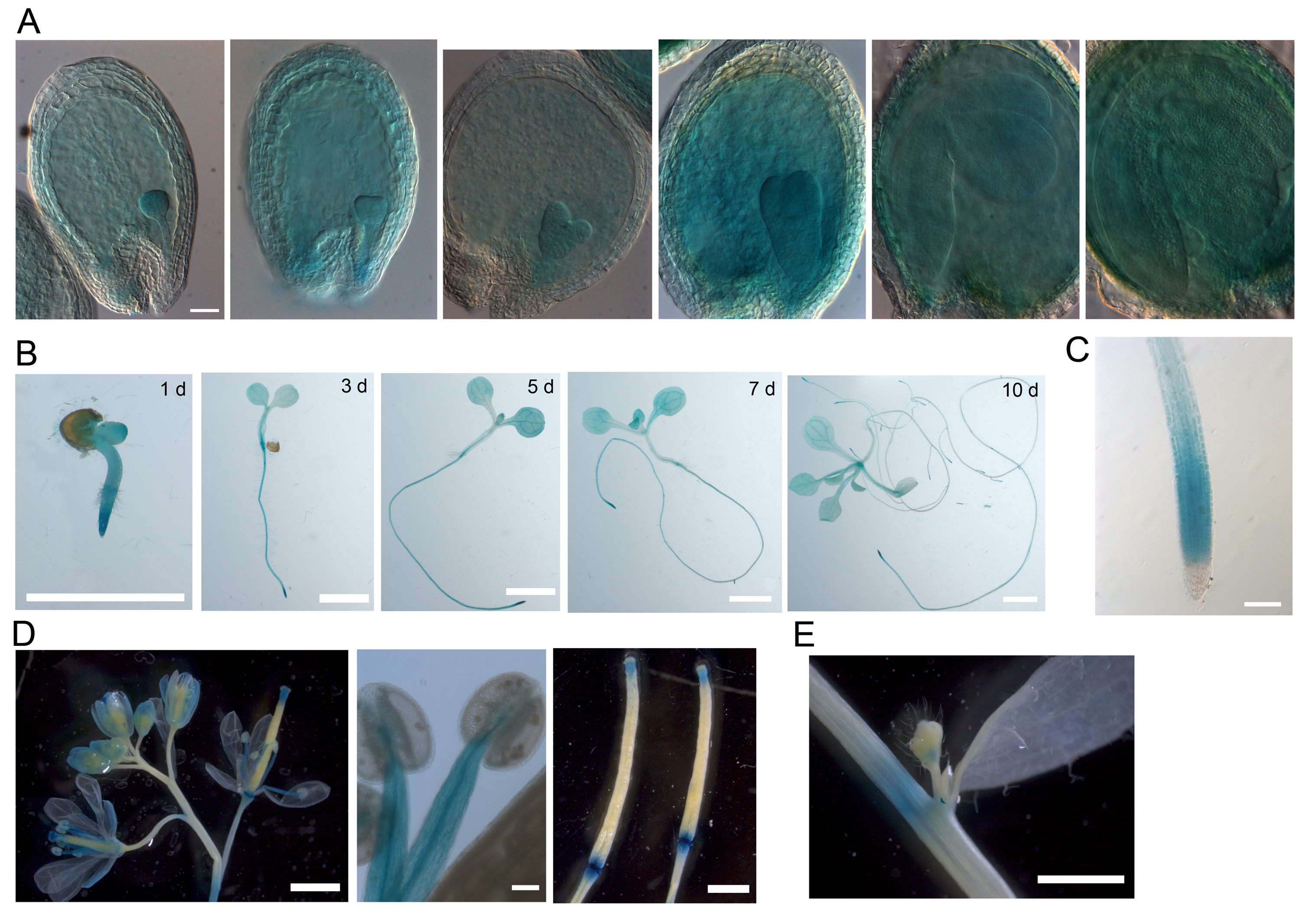

Supplement: S1 Fig — The tissue expression pattern of EMB1579 was revealed by monitoring the activity of GUS in transgenic plants harboring the fusion construct EMB1579pro-GUS. (A) Expression of EMB1579 in embryos at different stages. Bar = 50 μm. (B) Expression of EMB1579 in seedlings. Bars = 2 mm. (C) Expression of EMB1579 in root. Bar = 100 μm. (D) Expression of EMB1579 in flowers. The bar is 1.5 mm (left panel), 100 μm (middle panel), and 2 mm (right panel). (E) Expression of EMB1579 in a leaf branch. Bar = 1 mm. EMB1579, EMBRYO DEFECTIVE 1579. (TIF) [file pbio.3000782.s001.tif]

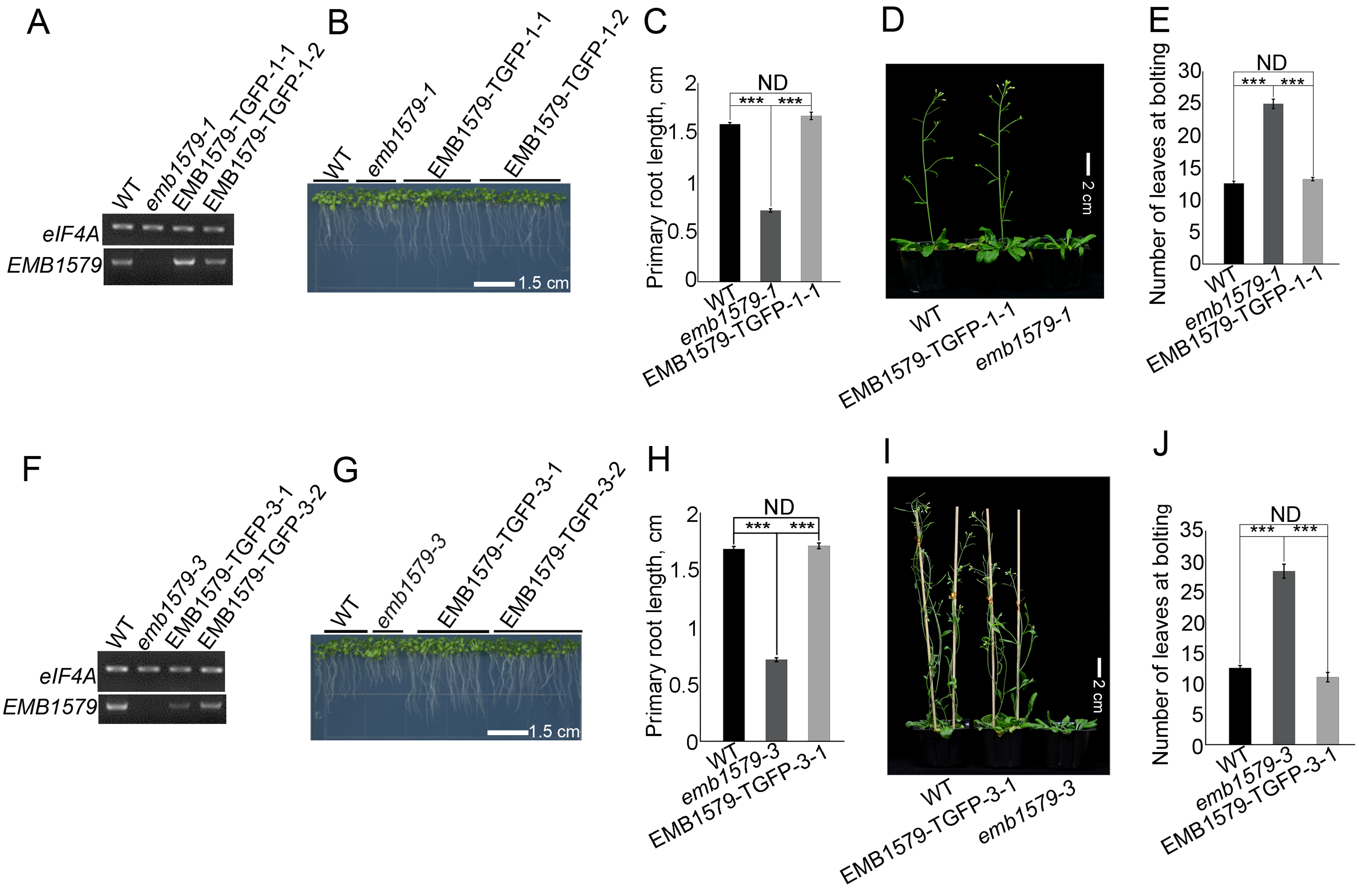

Supplement: S2 Fig — To complement emb1579 mutants, the construct pCAMBIA1301-proEMB1579:: gEMB1579-TGFP was transformed into emb1579-1 and emb1579-3 mutants. (A, F) RT-PCR analysis of the level of EMB1579 transcripts in WT, emb1579, and two complementation lines. The original pictures are available in S1 Raw Images. (B, G) Images of 7-day-old seedlings of WT, emb1579, and two complementation lines. Bars = 1.5 cm. (C, H) Quantification of primary root length of 7-day-old seedlings of WT, emb1579, and complementation lines. Data are presented as mean ± s.e.m. ***P < 0.001 by Student t test. Numerical data underlying the panels are available in S1 Data. (D) Images of 6-week-old Arabidopsis plants of WT, emb1579-1, and a complementation line. Bar = 2 cm. (E, J) Quantification of the number of rosette leaves at bolting in WT, emb1579, and complementation lines. Data are presented as mean ± s.e.m. ***P < 0.001 by Student t test. Numerical data underlying these panels are available in S1 Data. (I) Images of 7-week-old Arabidopsis plants of WT, emb1579-3, and a complementation line. Bar = 2 cm. emb1579, embryo defective 1579; ND, no significant difference; RT-PCR, reverse transcription PCR; WT, wild type. (TIF) [file pbio.3000782.s002.tif]

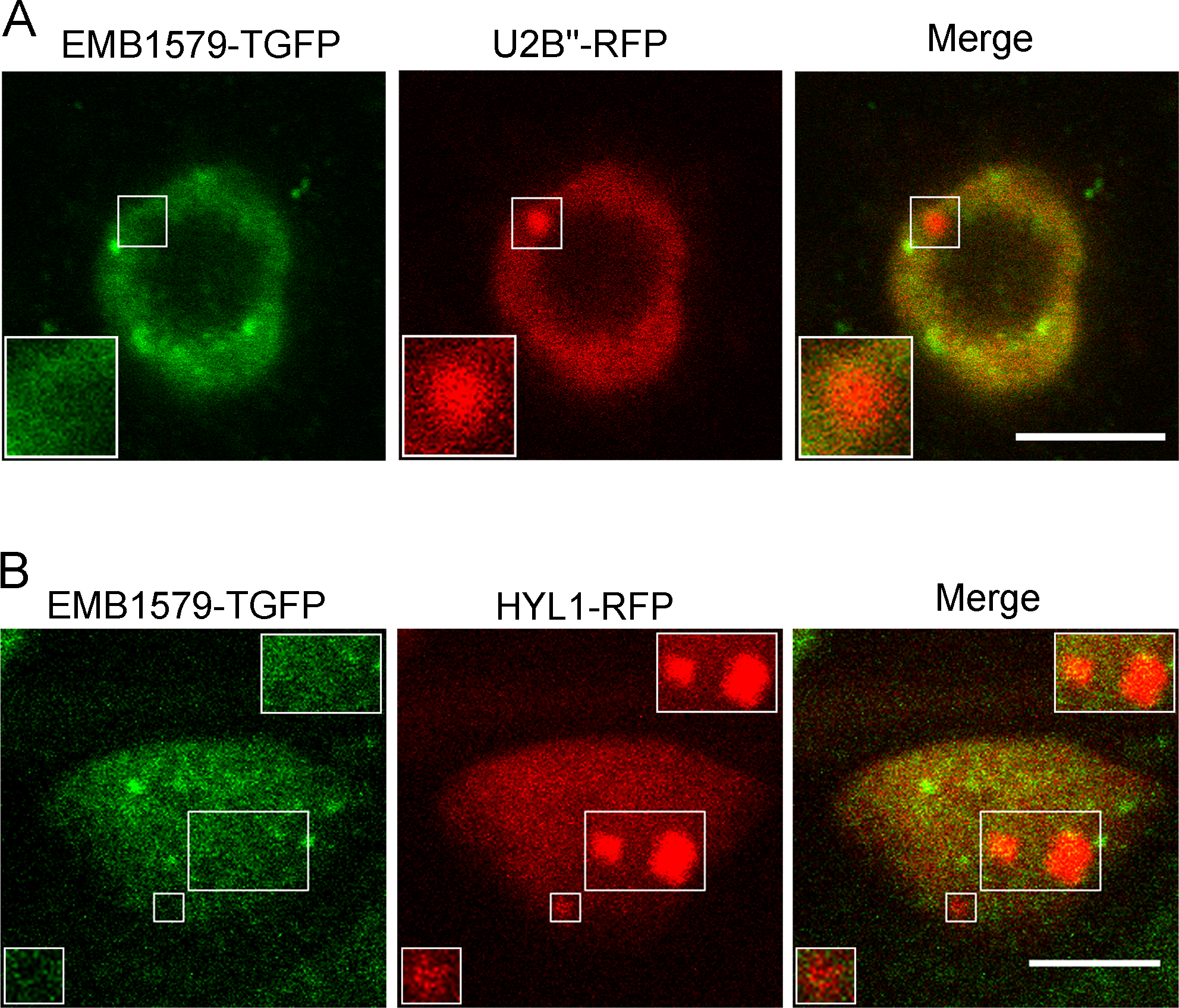

Supplement: S3 Fig — (A) Micrograph of Arabidopsis root cells expressing proEMB1579::gEMB1579-TGFP and 35S::U2B''-RFP. Bar = 5 μm. (B) Micrograph of Arabidopsis root cells expressing proEMB1579::gEMB1579-TGFP and 35S::HYL1-RFP. Bar = 5 μm. EMB1579, EMBRYO DEFECTIVE 1579. (TIF) [file pbio.3000782.s003.tif]

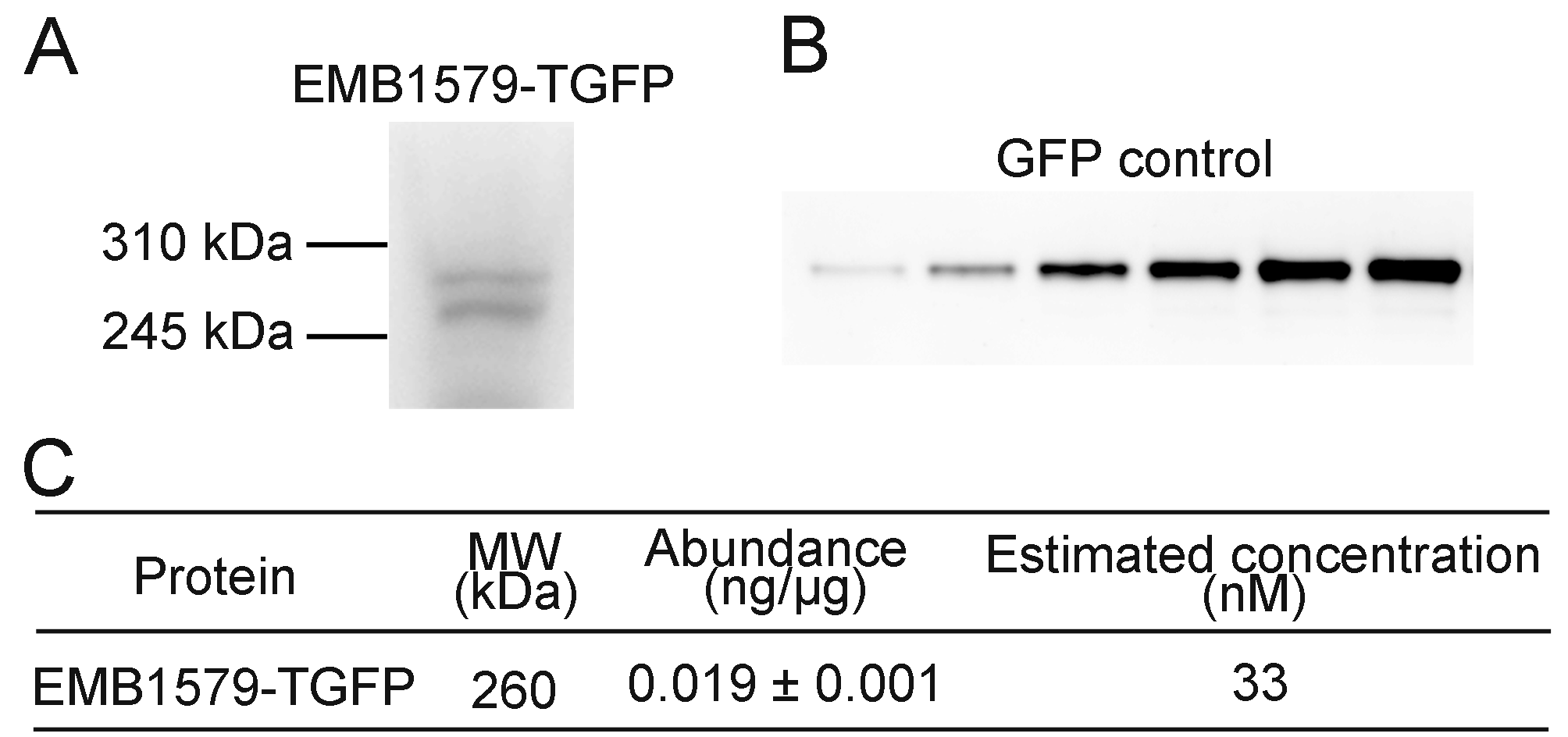

Supplement: S4 Fig — (A) Western blot analysis of nuclear proteins from proEMB1579::gEMB1579-TGFP; emb1579. The western blot was probed with anti-GFP antibody. The original pictures are available in S1 Raw Images. (B) Western blot analysis of recombinant GFP protein (1–10 ng), which was used as a loading control to quantify EMB1579-TGFP. The western blot was probed with anti-GFP antibody. The original pictures are available in S1 Raw Images. (C) Quantification of the concentration of EMB1579-TGFP in the nucleus. The abundance of EMB1579-TGFP protein was defined as the ratio of the amount of EMB1579-TGFP versus the amount of total protein. The value is presented as mean ± SD. EMB1579, EMBRYO DEFECTIVE 1579; GFP, green fluorescent protein; TGFP, tandem copies of enhanced GFP. (TIF) [file pbio.3000782.s004.tif]

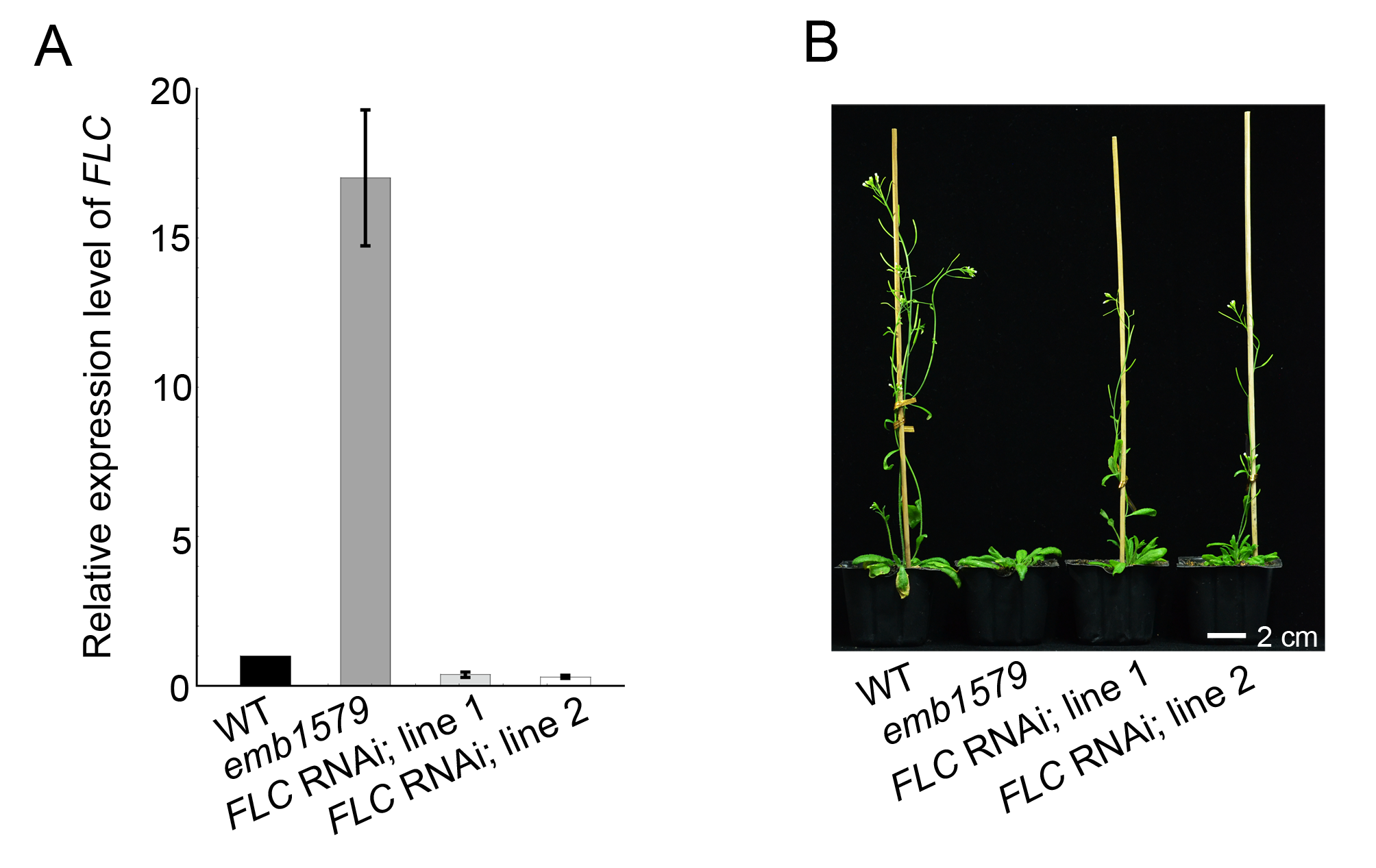

Supplement: S5 Fig — (A) RNAi-mediated knock-down of FLC in emb1579 plants. Relative expression of FLC was determined by qRT-PCR analysis. Data are presented as mean ± s.e.m, n = 3. FLC-RNAi plants are emb1579 plants expressing pFGC5941-FLC-RNAi. Numerical data underlying this panel are available in S1 Data. (B) Images of 6-week-old Arabidopsis plants. Bar = 2 cm. emb1579, embryo defective 1579; FLC, FLOWERING LOCUS C; qRT-PCR, quantitative reverse transcription PCR; RNAi, RNA interference. (TIF) [file pbio.3000782.s005.tif]

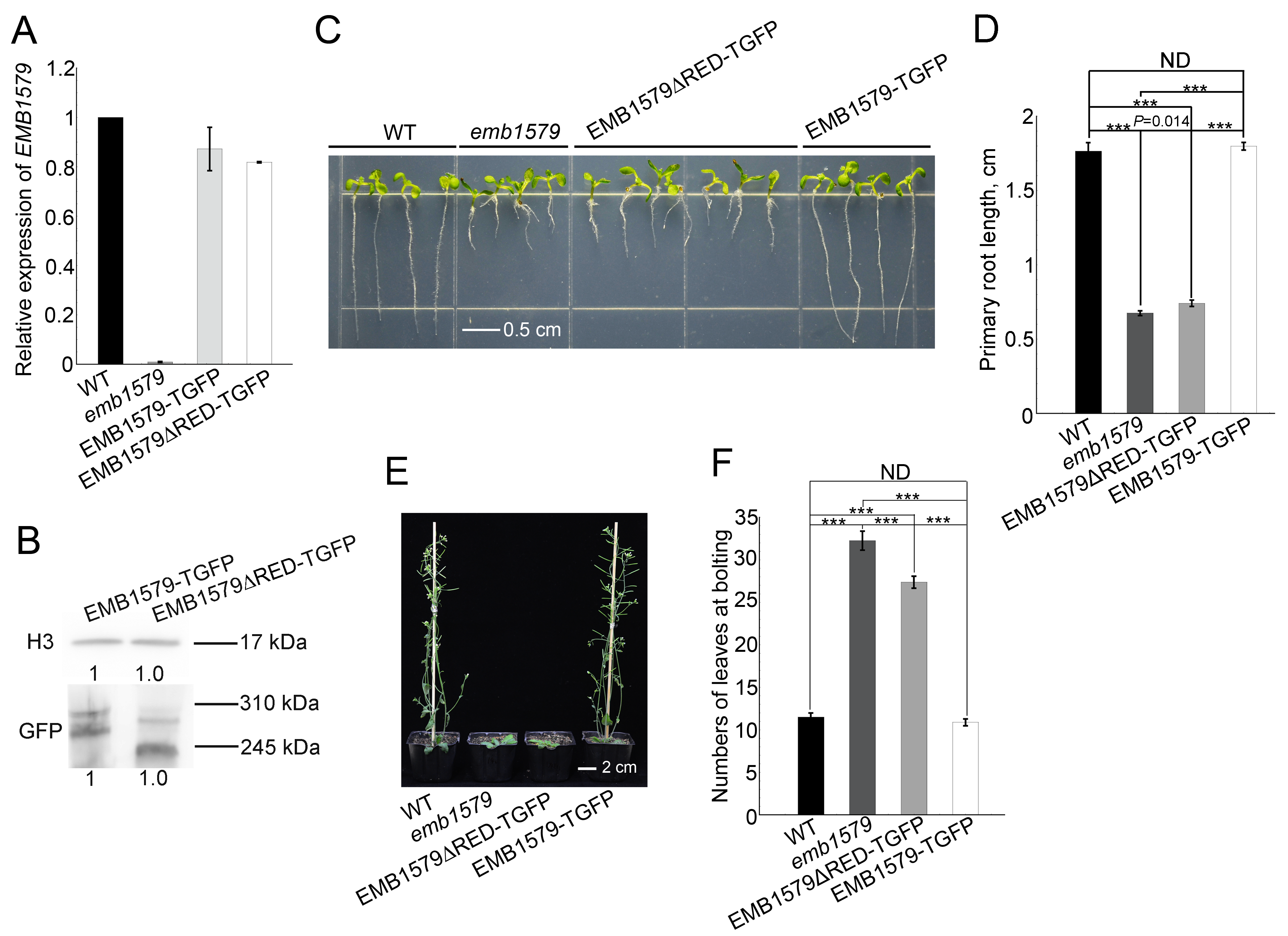

Supplement: S6 Fig — (A) qRT-PCR analysis to determine the relative level of EMB1579 transcripts in WT, emb1579, and the complementation plants. EMB1579-TGFP, expression of EMB1579-TGFP under control of the EMB1579 promoter in emb1579 mutants; EMB1579ΔRED-TGFP, expression of EMB1579ΔRED-TGFP under control of the EMB1579 promoter in emb1579 mutants. Data are presented as mean ± s.e.m, n = 3. Numerical data underlying this figure are available in S1 Data. (B) Western blot analysis to determine the relative amount of EMB1579-TGFP and EMB1579ΔRED-TGFP in the nucleus. Total nuclear proteins from Arabidopsis seedlings were probed with anti-GFP antibody. H3 protein (detected with an anti-H3 antibody) was used as the loading control. The original pictures are available in S1 Raw Images. (C) Images of 7-day-old Arabidopsis seedlings growing on plates. Bar = 0.5 cm. (D) Quantification of primary root length of 7-day-old seedlings in WT, emb1579, and its complementation lines. Data are presented as mean ± s.e.m. ***P < 0.001 by Student t test. Numerical data underlying this panel are available in S1 Data. (E) Images of 6-week-old Arabidopsis plants growing in pots. Bar = 2 cm. (F) Quantification of the number of rosette leaves at bolting in WT, emb1579, and the complementation lines. Data are presented as mean ± s.e.m. ***P < 0.001 by Student t test. Numerical data underlying this panel are available in S1 Data. EMB1579, EMBRYO DEFECTIVE 1579; ND, no significant difference; qRT-PCR, quantitative reverse transcription PCR; TGFP, tandem copies of enhanced green fluorescent protein; WT, wild type. (TIF) [file pbio.3000782.s006.tif]

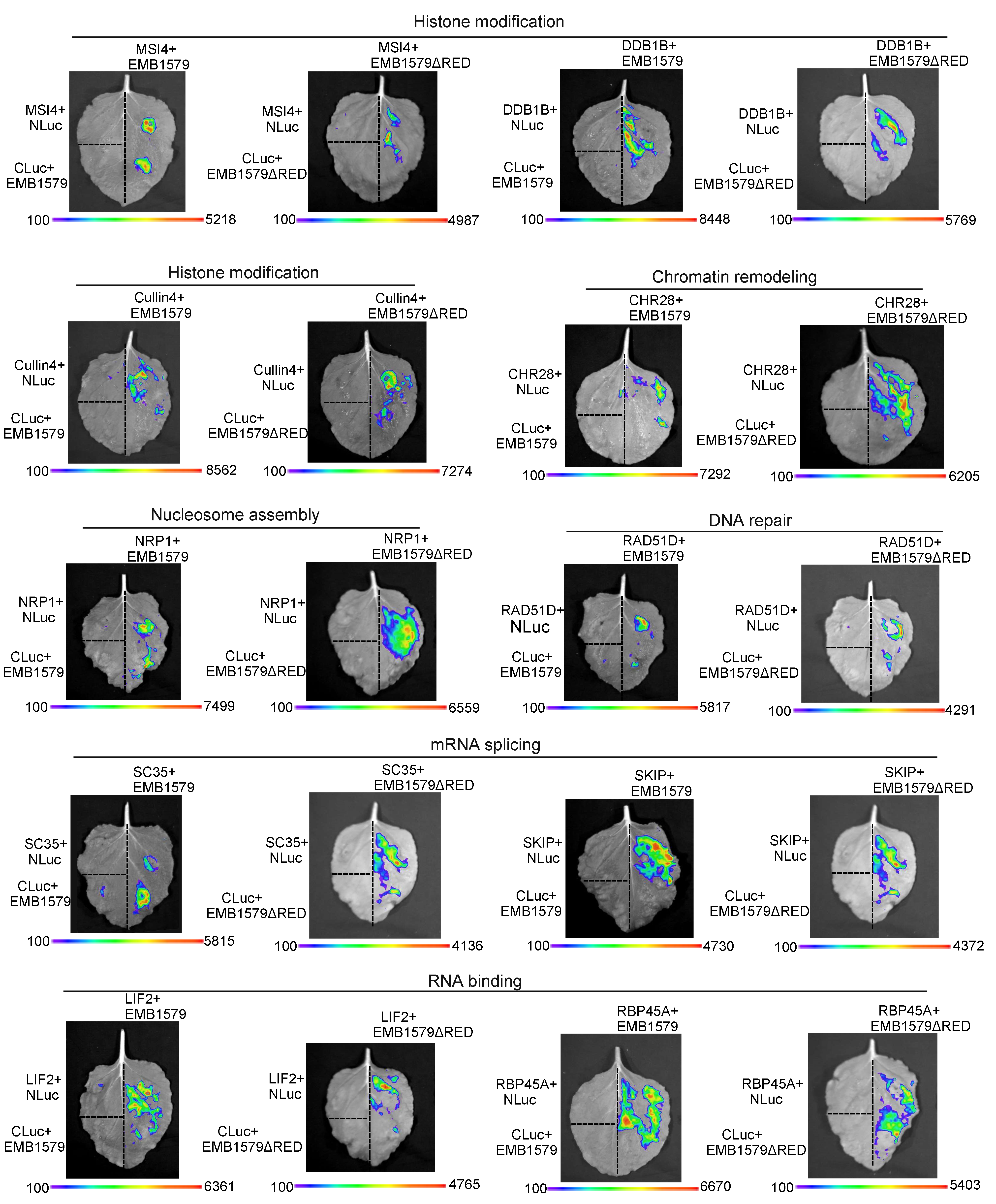

Supplement: S7 Fig — Interactions were detected by the firefly split luciferase complementation imaging assay. In total, 20 EMB1579-interacting proteins were tested for their interaction with EMB1579ΔRED. EMB1579, EMBRYO DEFECTIVE 1579. (TIF) [file pbio.3000782.s007.tif]

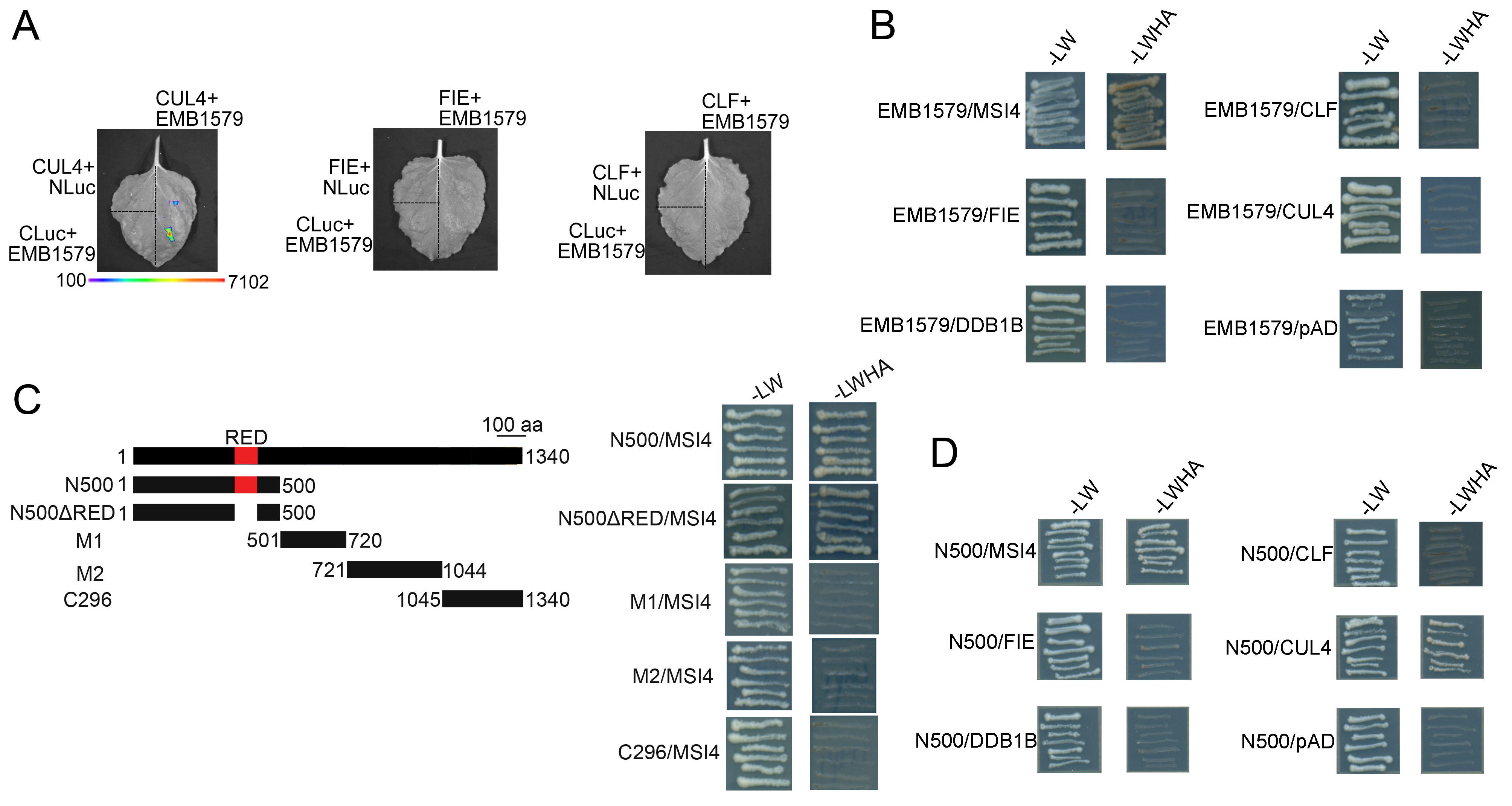

Supplement: S8 Fig — (A) The firefly split luciferase complementation imaging assay was used to determine the interactions of EMB1579 with CUL4, FIE, and CLF. (B) Yeast two-hybrid analysis of the interactions of EMB1579 with MSI4, CLF, FIE, CUL4, and DDB1B. (C) Mapping the binding region of MSI4 in EMB1579. The left panel shows schematic diagrams of EMB1579 and its truncations. The red box represents the RED repeat. The right panel shows yeast two-hybrid analysis of the interaction of the truncated EMB1579 proteins with MSI4. (D) Yeast two-hybrid analysis was performed to detect the interactions between N500 and MSI4, CLF, FIE, CUL4, or DDB1B. CLF, CURLY LEAF; CUL4, Cullin 4; DDB1, DNA Damage Binding Protein 1; EMB1579, EMBRYO DEFECTIVE 1579; FIE, FERTILIZATION INDEPENDENT ENDOSPERM; MSI4, MULTIPLE SUPPRESSOR OF IRA 4. (TIF) [file pbio.3000782.s008.tif]

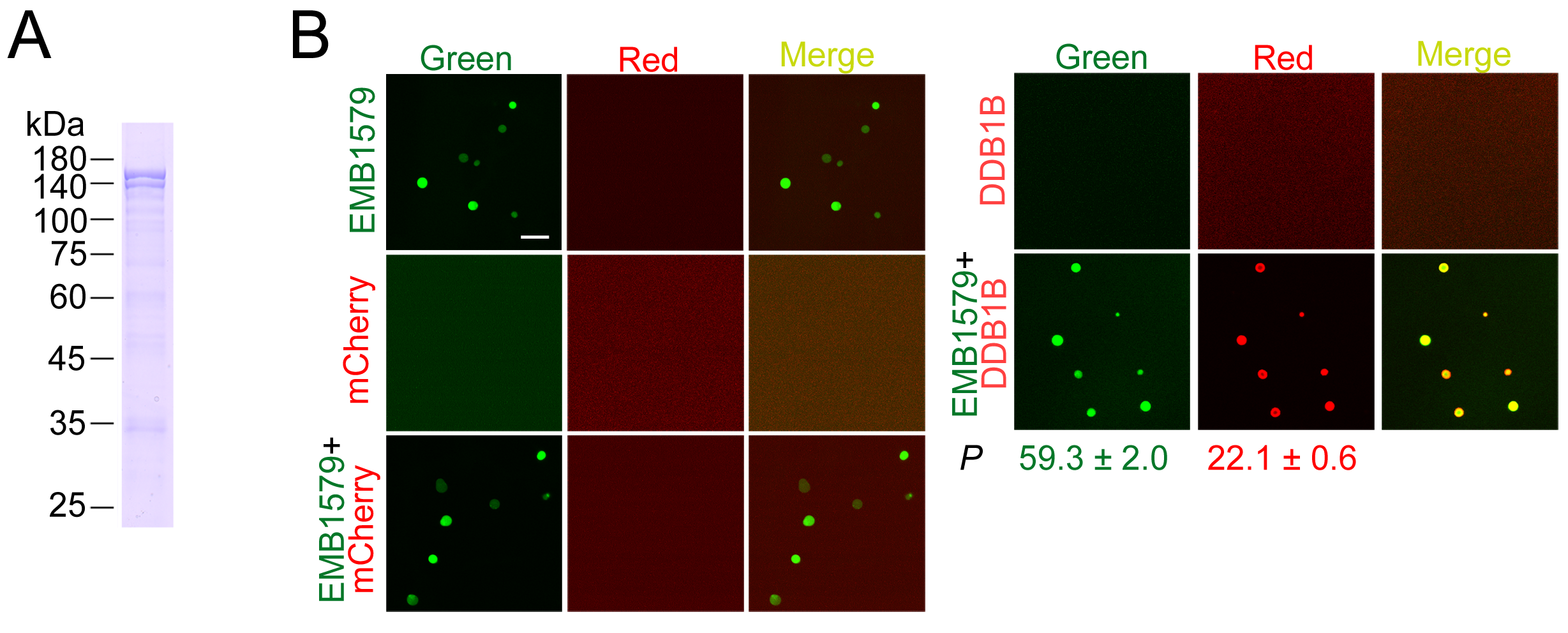

Supplement: S9 Fig — (A) SDS-PAGE analysis of recombinant mCherry-DDB1B. The original pictures are available in S1 Raw Images. (B) Visualization of DDB1B and EMB1579 in vitro under conditions that cause phase separation of EMB1579 (F-buffer: 25 mM Hepes [pH 8.0], 100 mM KCl, 100 mg/ml PEG 3350). mCherry, 12 μM; mCherry-DDB1B, 0.5 μM; EMB1579, 2.5 μM. The partition coefficient values were measured from 112 EMB1579 condensates and 112 DDB1 condensates. Data are presented as mean ± s.e.m. Bar = 10 μm. The underlying numerical data are available in S1 Data. DDB1, DNA Damage Binding Protein 1; EMB1579, EMBRYO DEFECTIVE 1579. (TIF) [file pbio.3000782.s009.tif]

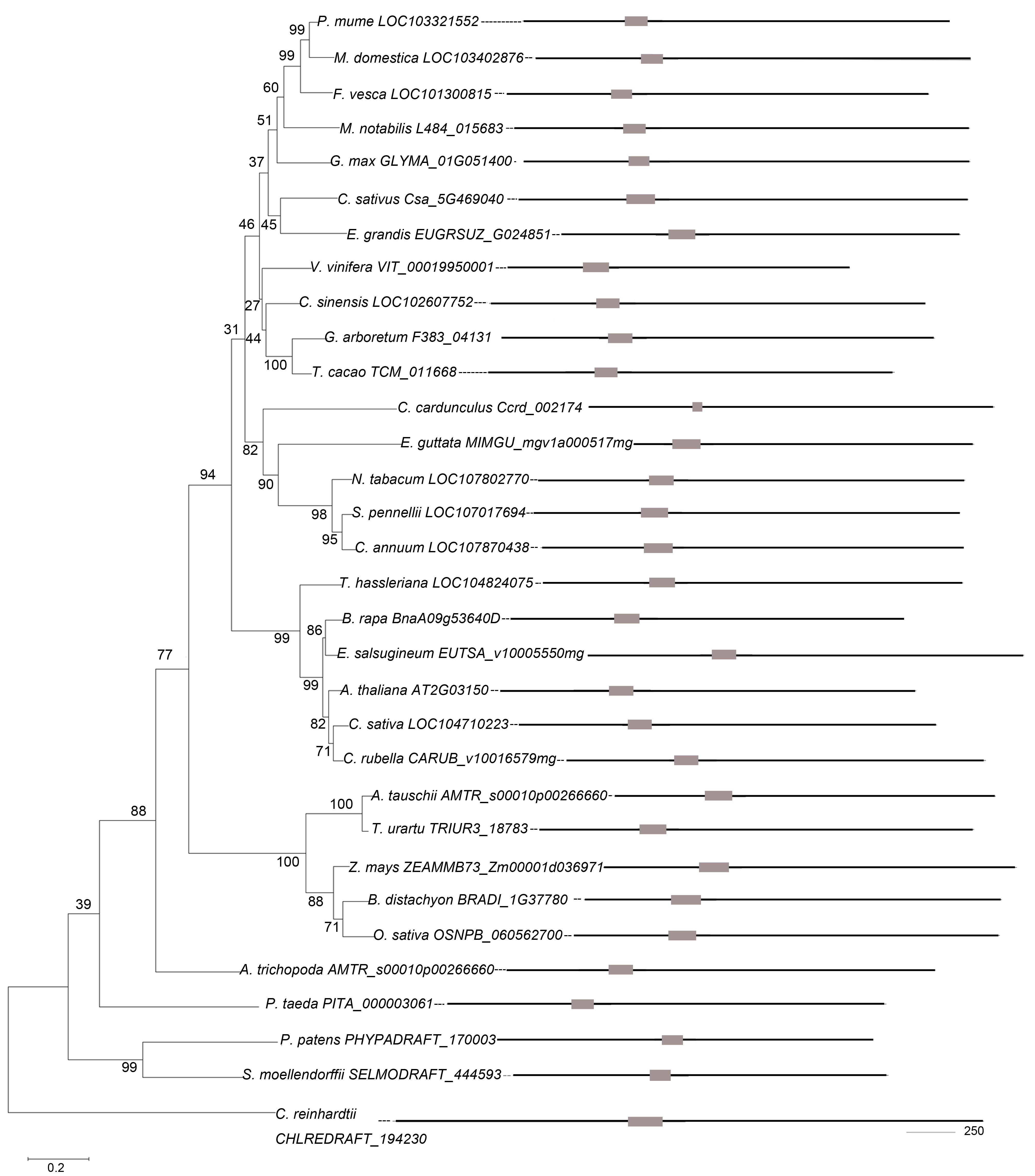

Supplement: S10 Fig — The phylogenetic tree of EMB1579 and its homologues was constructed with MEGA5.0 software. The accession numbers of EMB1579 and its homologues can be found either in GenBank or at the website http://congenie.org/ as follows: Arabidopsis thaliana, NP_178414; Aegilops tauschii, XP_020167627; Amborella trichopoda, XP_006827314; Brachypodium distachyon, XP_003563745; Brassica rapa, XP_009114177; Camelia sativa, XP_010425097; Capsella rubella, XP_006290321; Capsicum annuum, XP_016572460; Chlamydomonas reinhardtii, XP_001701130; Citrus sinensis, XP_006483121; Cucumis sativus, XP_011655281; Cynara cardunculus, KVH95716; Erythranthe guttata, EYU30242; Eucalyptus grandis, XP_010066890; Eutrema salsugineum, XP_006395736; Fragaria vesca, XP_004297287; Glycine max, XP_006573124; Gossypium arboretum, KHG15037; Malus domestica, XP_008339877; Morus notabilis, XP_010108695; Nicotiana tabacum, XP_016481812; Oryza sativa, XP_015643243; Physcomitrella patens, XP_001777802; Picea taeda, PITA_000003061; Prunus mume, XP_016647712; Selaginella moellendorffii, XP_002980580; Solanum pennellii, XP_015073405; Tarenaya hassleriana, XP_010554305; Theobroma cacao, EOY01867; Triticum urartu, EMS67387; Vitis vinifera, XP_010651850; Zea mays, XP_008649139. The gray boxes represent conserved RED repeats. EMB1579, EMBRYO DEFECTIVE 1579. (TIF) [file pbio.3000782.s010.tif]

Fig 1A

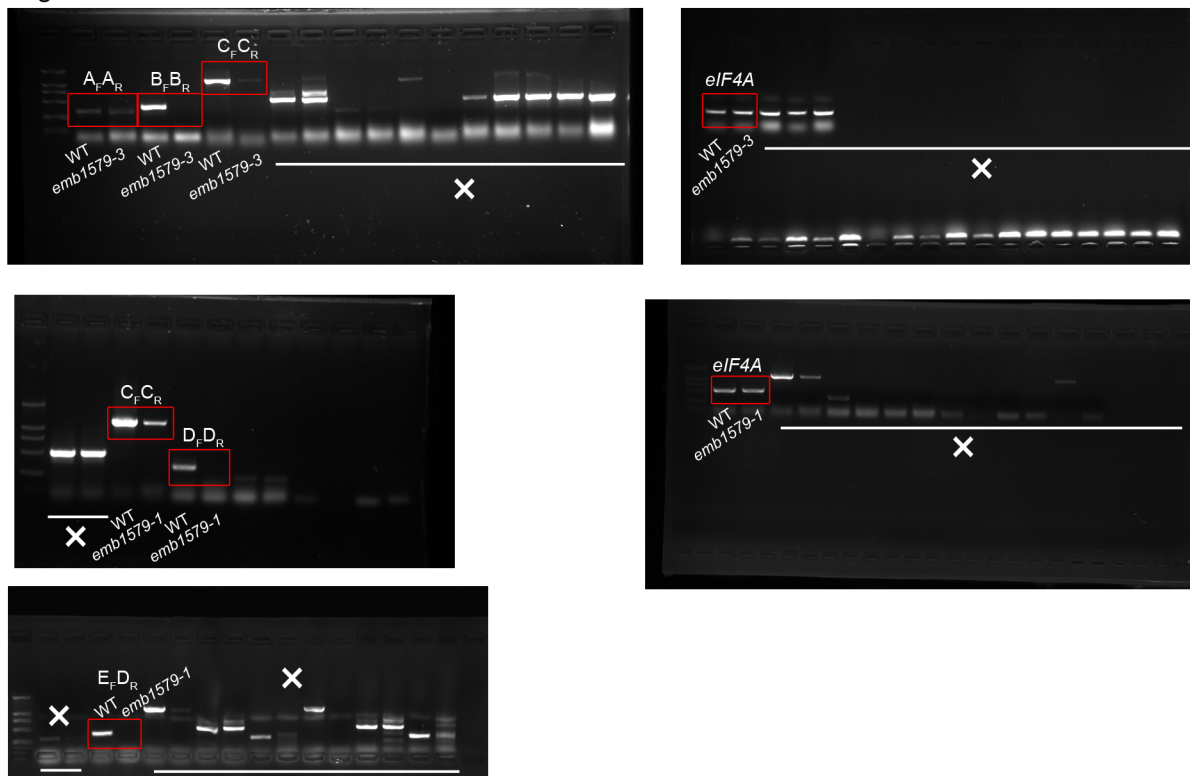

Fig 2J

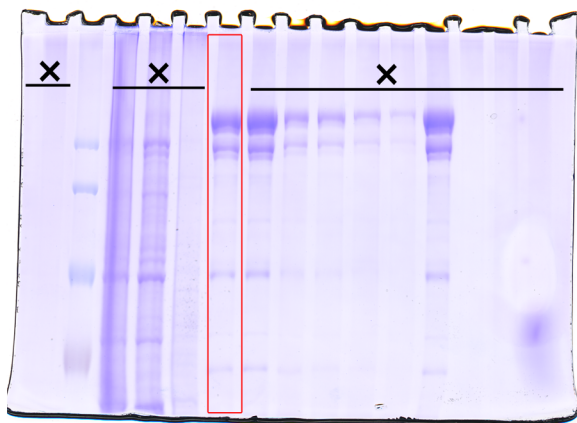

Fig 5B

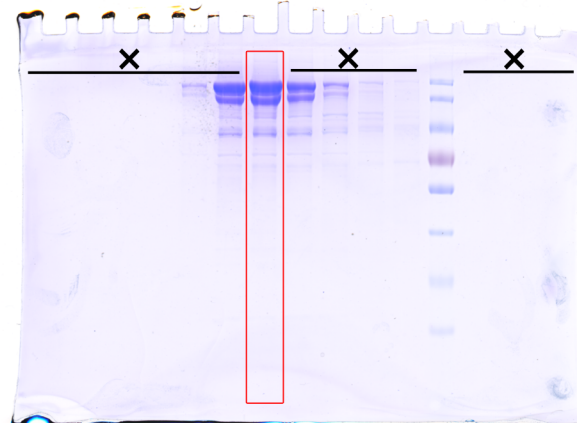

Fig 6D

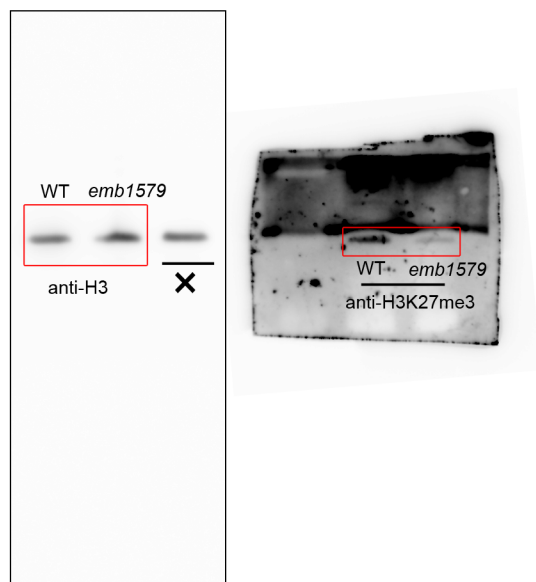

S2A & S2F Fig

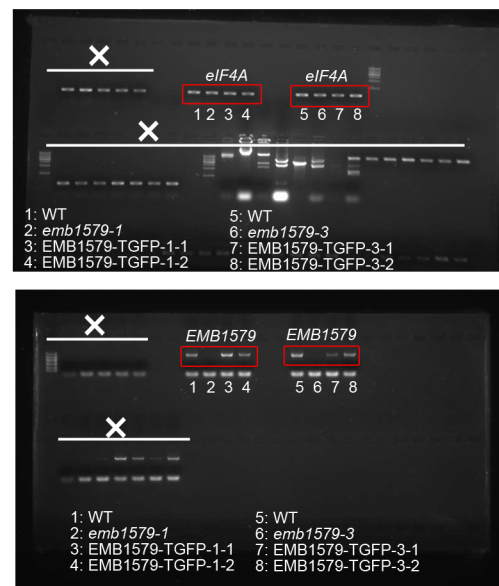

S4A Fig

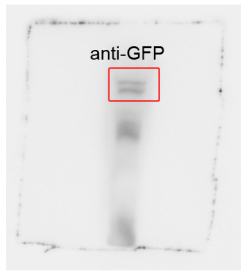

S4B Fig

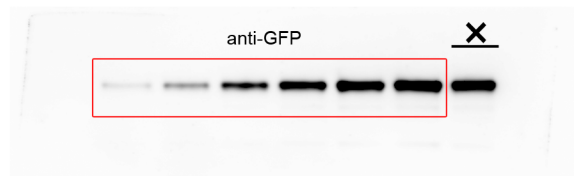

S6B Fig

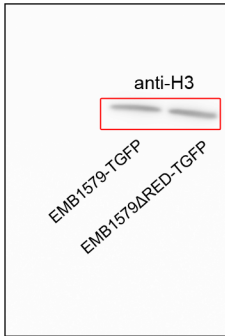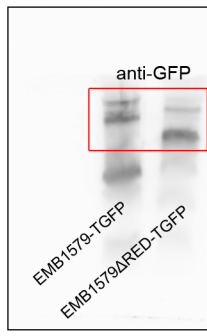

Fig 6F & S9A Fig

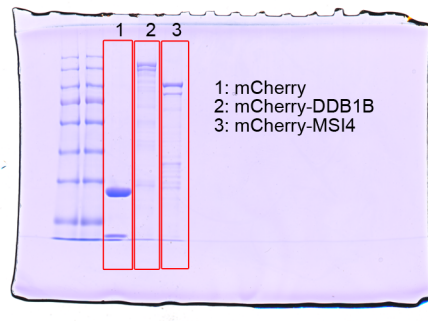

Supplement: S1 Raw Images — (PDF) [file pbio.3000782.s022.pdf]
