# Supplementary material for: Phase separation of Arabidopsis EMB1579 controls transcription, mRNA splicing, and development
Source: PLoS Biol. 2020 Jul 21;18(7):e3000782. doi: 10.1371/journal.pbio.3000782 (PMC7413564; doi:10.1371/journal.pbio.3000782)
Supplement: S4 Table — (DOC) [file pbio.3000782.s019.doc]

**S4 Table. Primers used in this study**

| **Primer Name** | **Primer Sequence (5' to 3')** |
| --- | --- |
| Salk_007142-LP | CAAAGGTCCTGAGAGAACAGATC |
| Salk_007142-RP | CGTGAAAATTTCGGTTGATTC |
| CS16026-LP | TCATGTCTCTTCTTCAGCCGC |
| CS16026-RP | TTCATGTTGTTTCCTTTTCTCACAA |
| Salk LB | ATTTTGCCGATTTCGGAAC |
| Sail LB | TAGCATCTGAATTTCATAACCAATCTCGATACAC |
| Sail_1167_E05-LP | CCCACGAGGTGTATATCATGG |
| Sail_1167_E05-RP | TTACCTGCAATGTTCCACCTC |
| proEMB1579-SalI-F | **GTCGAC**ACAATAAGATGTTGGAGTTTTC |
| proEMB1579-BamHI-R | C**GGATCC**CTGGTTTCAATTTTGTAATTTTA |
| gEMB1579-SmaI-F | GA**CCCGGG**TAATGCATAGAGATATGTATTCTTCA |
| gEMB1579-SacI-R | GA**GAGCTC**TAGAGACAATCTAACAAGTTTATTG |
| ΔRED-F | CGGACTCCAACAGCAAGAGCTACGTCC |
| ΔRED-R | GAGATAACTTGCCCGGTCACCTTTCCG |
| 35S-HindIII-F | TGC**AAGCTT**GTATTGGCTAGAGCAGCTTGAGCTTG |
| 35S-PstI-R | ATA**CTGCAG**AGAGATAGATTTGTAGAGAGAGACTGG |
| GFP-NLS-SacI-F | CC**GAGCTC**ATGGTGAGCAAGGGCGAGGAGCTGTTC |
| GFP-NLS-EcoRI-R | CC**GAATTC**TTAAACTTTTCTTTTTTTTTTAGGCTTGTACAGCTCGTCCATGCCGAGAGTG |
| NOS-EcoRI-F | CC**GAATTC**CGTTCAAACATTTGGCAATAAAGTTTC |
| NOS-BstXI-R | AGT**CCAACATGGTGG**CCGATCTAGTAACATAGATGACACCGCGC |
| GUS-BamHI-R | **GGATCC**ATTTTGCAACTCCCTACCGA |
| U2B''-SalI-F | CC**GTCGAC**ATGTTAACGGCAGATATACCACCGAATC |
| U2B''-KpnI-R | AA**GGTACC**TTTCTTGGCGAAAGAGATGACCATTGG |
| RFP-BamHI-linker-F | AA**GGATCC**GGTGGAGGTGGAGCCTCCTCCGAGGACGTC |
| RFP-HindIII-R | AGC**AAGCTT**TTAGGCGCCGGTGGAGTGGCGGCC |
| U1-70K-AscI-F | *ATTTACAATTACCATGG***GGCGCGCC**ATGGGAGACTCCGGCGATCCTTTCTTGC |
| U1-70K-linker-R | *TGATGACGTCCTCGGAGGAGGCCAT*ACCAGCACCAGCACCAGCACCACGAACATACTCTCGCGATTCTGATCTCTT |
| HYL1-AscI-F | *ATTTACAATTACCATGG***GGCGCGCC**ATGACCTCCACTGATGTTTCCTCTGGTG |
| HYL1-linker-R | *TGATGACGTCCTCGGAGGAGGCCAT*ACCAGCACCAGCACCAGCACCTGCGTGGCTTGCTTCTGTCTCCACACTCCC |
| SC35-AscI-F | *ACATTTACAATTACCATGG***GGCGCGCC**ATGTCGCACTTCGGAAGGTCAGGTCCAC |
| SC35-linker-R | *TGATGACGTCCTCGGAGGAGGCCAT*ACCAGCACCAGCACCAGCACCTTCCGCAGCATAAGGAGATTGACTTCGAG |
| DDB1B-AscI-F | *ACATTTACAATTACCATGG***GGCGCGCC**ATGAGCGTATGGAACTACGCCGTTACGGC |
| DDB1B-RFP-R | *TGATGACGTCCTCGGAGGAGGCCAT*ACCAGCACCAGCACCAGCACCGTGAAGCCTAGTGAGTTCTTCAACTCTCTT |
| RFP-RZ-1C-SwaI-F | *GCGCCGGTGGAGGTGGAC***ATTTAAAT**ATGGCTGCAAAAGAAGGTAGTAGGATTT |
| RFP-RZ-1C-PacI-R | *TAGGGACTAGTCCCGGGTC***TTAATTAA**TTAATAACGGTCAAAAGTGGACGACGACG |
| RFP-MSI4-SwaI-F | *GCGCCGGTGGAGGTGGAC***ATTTAAAT***ATGGAGAGCGACGAAGCAGCAGCAGTG* |
| RFP-MSI4-PacI-R | *TAGGGACTAGTCCCGGGTC***TTAATTAA**TTAAGGCTTGGAGGCACAAGTCATAACATG |
| U1-70K-linker-RFP-F | *ATCAGAATCGCGAGAGTATGTTCGT*GGTGCTGGTGCTGGTGCTGGTATGGCCTCCTCCGAGGACGTCATCAAGG |
| linker-RFP-PacI-R | *GGGACTAGTCCCGGGTC***TTAATTAA**TTAGGCGCCGGTGGAGTGGCGGCCCTCG |
| HYL1-linker-RFP-F | *TGTGGAGACAGAAGCAAGCCACGCA*GGTGCTGGTGCTGGTGCTGGTATGGCCTCCTCCGAGGACGTCATCAAGG |
| SC35-linker-RFP-F | *GAAGTCAATCTCCTTATGCTGCGGAA*GGTGCTGGTGCTGGTGCTGGTATGGCCTCCTCCGAGGACGTCATCAAGG |
| DDB1B-linker-RFP-F | *GAGTTGAAGAACTCACTAGGCTTCAC*GGTGCTGGTGCTGGTGCTGGTATGGCCTCCTCCGAGGACGTCATCAAGG |
| RFP-AscI-F | A**GGCGCGCC**ATGGCCTCCTCCGAGGACGTC |
| RFP-SwaI-R | TT**ATTTAAAT**GTCCACCTCCACCGGCGCCGGTGGAGTGGCG |
| GFP-EcoRI-R | CC**GAATTC**TTACTTGTACAGCTCGTCCATGCCGAGAGT |
| EMB1579-KpnI-F | *GAACACGGGGGACGAGCTC***GGTACC**ATGCATAGAGATATGTATTCTTCAAG |
| EMB1579-SalI-R | *GGGACGCGTACGAGATCTG***GTCGAC**TAGAGACAATCTAACAAGTTTATTG |
| MSI4-KpnI-F | *CTCGTACGCGTCCCGGGGC***GGTACC**ATGGAGAGCGACGAAGCAGCAGC |
| MSI4-SalI-R | *ACGAACGAAAGCTCTGCAG***GTCGAC**TTAAGGCTTGGAGGCACAAGTCATAAC |
| DDB1B-KpnI-F | *CTCGTACGCGTCCCGGGGC***GGTACC**ATGAGCGTATGGAACTACGCCGTTAC |
| DDB1B-SalI-R | *ACGAACGAAAGCTCTGCAG***GTCGAC**TCAGTGAAGCCTAGTGAGTTCTTCAAC |
| CUL4-KpnI-F | *CTCGTACGCGTCCCGGGGC***GGTACC**ATGTCTCTTCCTACCAAACGCTCTAC |
| CUL4-SalI-R | *ACGAACGAAAGCTCTGCAG***GTCGAC**CTAAGCAAGATAATTGTATATCTGAGG |
| FIE-KpnI-F | *CTCGTACGCGTCCCGGGGC***GGTACC**ATGTCGAAGATAACCTTAGGGAAC |
| FIE-SalI-R | *ACGAACGAAAGCTCTGCAG***GTCGAC**CTACTTGGTAATCACGTCCCAGCG |
| CLF-KpnI-F | *CTCGTACGCGTCCCGGGGC***GGTACC**ATGGCGTCAGAAGCTTCGCCTTCTTC |
| CLF-SalI-R | *ACGAACGAAAGCTCTGCAG***GTCGAC**CTAAGCAAGCTTCTTGGGTCTACC |
| CDKC;2- KpnI-F | *CTCGTACGCGTCCCGGGGC***GGTACC**ATGGCGGCTGCGGCTTTTGGGCAG |
| CDKC;2 -SalI-R | *ACGAACGAAAGCTCTGCAG***GTCGAC**TTACGGTTGCCATCCATATTGTTGG |
| SKIP-KpnI-F | *CTCGTACGCGTCCCGGGGC***GGTACC**ATGGTGACAGACAAGAGCAAGAAG |
| SKIP-SalI-R | *ACGAACGAAAGCTCTGCAG***GTCGAC**TCATTCCTCACCAACTTCGTCTTC |
| UAP56A-KpnI-F | *CTCGTACGCGTCCCGGGGC***GGTACC**ATGGGAGACGCTAGAGACAACGAAG |
| UAP56A-SalI-R | *ACGAACGAAAGCTCTGCAG***GTCGAC**TTAAGAAGGCATGTAGGTTGAAGTATC |
| SC35-KpnI-F | *CTCGTACGCGTCCCGGGGC***GGTACC**ATGTCGCACTTCGGAAGGTCAGGTC |
| SC35SalI-R | *ACGAACGAAAGCTCTGCAG***GTCGAC**TCATTCCGCAGCATAAGGAGATTGAC |
| U1-70K-KpnI-F | *CTCGTACGCGTCCCGGGGC***GGTACC**ATGGGAGACTCCGGCGATCCTTTC |
| U1-70K-SalI-R | *ACGAACGAAAGCTCTGCAG***GTCGAC**TCAACGAACATACTCTCGCGATTC |
| RZ-1C-KpnI-F | *CTCGTACGCGTCCCGGGGC***GGTACC**ATGGCTGCAAAAGAAGGTAGTAGG |
| RZ-1C-SalI-R | *ACGAACGAAAGCTCTGCAG***GTCGAC**TTAATAACGGTCAAAAGTGGACGAC |
| EBP1-KpnI-F | *CTCGTACGCGTCCCGGGGC***GGTACC**ATGAGTTCGGACGATGAGAGAGACG |
| EBP1-SalI-R | *ACGAACGAAAGCTCTGCAG***GTCGAC**TCATTCTTGAGCATTACTACTTGCG |
| RBP47C-KpnI-F | *ATCTCGTACGCGTCCCGGGGC***GGTACC**ATGGCAGACGTCAAGGTTCAATCC |
| RBP47C-SalI-R | *ATACGAACGAAAGCTCTGCAG***GTCGAC**TCAGCTAACTTGTTGCTGATGACCAC |
| LIF2-KpnI-F | *ATCTCGTACGCGTCCCGGGGC***GGTACC**ATGTCAGACGCAAGAGATAATGATG |
| LIF2-SalI-R | *ATACGAACGAAAGCTCTGCAG***GTCGAC**CTAGTAGGGTCGGTACCTTCGGCCTC |
| RBP45A-KpnI-F | *ATCTCGTACGCGTCCCGGGGC***GGTACC**ATGCAGCAACCACCGTCAAACGCC |
| RBP45A-SalI-R | *ATACGAACGAAAGCTCTGCAG***GTCGAC**TCACTGACGTTGCTGCTGATAGTTGC |
| BTF3-KpnI-F | *CTCGTACGCGTCCCGGGGC***GGTACC**ATGAATAGGGAGAAGTTGATGAAG |
| BTF3-SalI-R | *ACGAACGAAAGCTCTGCAG***GTCGAC**CTAAGAAGAAGCAGCAGCAGCTGG |
| MED8-KpnI-F | *ATCTCGTACGCGTCCCGGGGC***GGTACC**ATGGAGACACAGCCGCAGCAACCAC |
| MED8-SalI-R | *ATACGAACGAAAGCTCTGCAG***GTCGAC**TTATTGAGGATTTTGGTGCCTTTGTTG |
| RAD23C-KpnI-F | *CTCGTACGCGTCCCGGGGC***GGTACC**ATGAAGATATTTGTGAAAACTCTC |
| RAD23C-SalI-R | *ACGAACGAAAGCTCTGCAG***GTCGAC**TTATTCCTCGAATTCATGCATGTGA |
| RAD51D-KpnI-F | *TCTCGTACGCGTCCCGGGGC***GGTACC**ATGGCGCCTCTCAAACATCTGGAGAAG |
| RAD51D-SalI-R | *TACGAACGAAAGCTCTGCAG***GTCGAC**TTATGGACATTGTTGATTCTCCTTGC |
| NRP1-KpnI-F | *ATCTCGTACGCGTCCCGGGGC***GGTACC**ATGGTCGCGGACAAGAGCAAGAAGTC |
| NRP1-SalI-R | *ATACGAACGAAAGCTCTGCAG***GTCGAC**TTACACATCTGTTATAGAAGCAAGCC |
| CHR28-KpnI-F | *ATCTCGTACGCGTCCCGGGGC***GGTACC**ATGGATTCCGCTATTGATATCAGTTC |
| CHR28-SalI-R | *ATACGAACGAAAGCTCTGCAG***GTCGAC**CTACACCATGAATAGATATTTGAGATC |
| MSI4-KpnI-F2 | AA**GGTACC**GAGAGCGACGAAGCAGCAGCAGTGTC |
| MSI4-XbaI-R | AC**TCTAGA**TTAAGGCTTGGAGGCACAAGTCATAAC |
| RFP-SalI-F | CC**GTCGAC**ATGGCCTCCTCCGAGGACGTCATCAAG |
| RFP-KpnI-R | AA**GGTACC**GGCGCCGGTGGAGTGGCGGCCCTCGG |
| FLC-RNAi-XbaI-AscI-F | AA**TCTAGAGGCGCGCC**CCTGGTCAAGATCCTTGATC |
| FLC-RNAi-BamHI-SwaI-R | GC**GGATCCATTTAAAT**CTATCCACAAGTTCAAGTAG |
| EMB1579-EcoRI-F | CG**GAATTC**ATGCATAGAGATATGTATTC |
| EMB1579-SalI-R2 | ACGC**GTCGAC**CTAATGGTGATGGTGATGGTGTAGAGACAATCTAACAAG |
| GFP-SaII-F | ACGC**GTCGAC**ATGGTGAGCAAGGGCGAGGAGCTG |
| GFP-XbaI-R | TGC**TCTAGA**TTAATGGTGATGGTGATGGTGCTTGTACAGCTCGTCCATGCCGAG |
| mCherry-NdeI-F | AA**CATATG**ATGGTGAGCAAGGGCGAGGAGGATAAC |
| mCherry-linker-KpnI-R | GG**GGTACC**TGCCGATCCGCCTGACGATCCGCCTGAGCCTCCCGACTTGTACAGCTCGTCCATGCCGCCGG |
| MSI4-SalI-F | AA**GTCGAC**TGCCGATCCGCCTGACGATCCGCCTGAGCCTCCCGACTTGTACAGCTCGTCCATGCCGCCGG |
| MSI4-XbaI-R2 | TT**TCTAGA**TTAATGGTGATGGTGATGGTGAGGCTTGGAGGCACAAGTCATAAC |
| mCherry-XhoI-F | ATT**CTCGAG**ATGGTGAGCAAGGGCGAGGAGGATAAC |
| mCherry-linker-EcoRI-R | AA**GAATTC**TGCCGATCCGCCTGACGATCCGCCTGAGCCTCCCGACTTGTACAGCTCGTCCATGCCGCCGGTG |
| DDB1B-EcoRI-F | AA**GAATTC**ATGAGCGTATGGAACTACGCCGTTAC |
| DDB1B-SalI-R2 | AA**GTCGAC**TTAATGGTGATGGTGATGGTGGTGAAGCCTAGTGAGTTCTTCAACTCTC |
| EMB1579-SfiI-F | AA**GGCCATTACGGCC**ATGCATAGAGATATGTATTCTTCAAGAG |
| EMB1579-SfiI-R | CC**GGCCGAGGCGGCC**CTATAGAGACAATCTAACAAGTTTATTG |
| N500-SalI-R | AA**GTCGAC**CTACACGTAATCCCTCTCCATGTC |
| M1-EcoRI-F | G**GAATTC**ACACTAGATAAAAGATATCCCAGGCTT |
| M1-SalI-R | AA**GTCGAC**CTACTTAACCTCCTGAGAGAGTAGTCGATC |
| M2-EcoRI-F | G**GAATTC**AAAGATACTGTTGAAGTAACCAAGGATG |
| M2-SalI-R | AA**GTCGAC**CTACATTTGATACTGCAGCATCTCATATA |
| C296-EcoRI-F | G**GAATTC**GGTTCCCGTATCTTTGAATTTCTCAAA |
| C296-SalI-R | AA**GTCGAC**CTATAGAGACAATCTAACAAGTTTATT |
| DDB1B-XhoI-R | CCG**CTCGAG**TCAGTGAAGCCTAGTGAGTTCTTCAAC |
| MSI4-NdeI-F | GGAATTC**CATATG**ATGGAGAGCGACGAAGCAGCAGC |
| MSI4-XhoI-R | CCG**CTCGAG**TTAAGGCTTGGAGGCACAAGTC |
| CUL4-EcoRI-F | G**GAATTC**ATGTCTCTTCCTACCAAACGCTCTAC |
| CUL4-BamHI-R | CG**GGATCCC**TAAGCAAGATAATTGTATATCTG |
| FIE-BamHI-F | CG**GGATCC**ATATGTCGAAGATAACCTTAGGGAACGAG |
| FIE-SacI-R | TT**GAGCTC**CTACTTGGTAATCACGTCCCAGCGCC |
| CLF-NdeI-F | TAT**CATATG**ATGGCGTCAGAAGCTTCGCCTTCTTC |
| CLF-EcoRI-R | G**GAATTC**CTAAGCAAGCTTCTTGGGTCTACCAAC |
| EMB1579-C_F_-RT | ATCCCAGTGCTAAGGCTAATG |
| EMB1579-C_R_-RT | CAGTGCCACTAGTTTTCTCAAG |
| EMB1579-D_F_-RT | AGGCAGGCTATGTTAGGGTT |
| EMB1579-D_R_-RT | CGCAAGATTACTGGTTATTGTAC |
| EMB1579-E_F_-RT | TTAGAGAGCAACACTGGAAGAG |
| EMB1579-A_F_-RT | AGCAAACTAATCCTCAAACCA |
| EMB1579-A_R_-RT | TGGATGGCGAGAACTCAAAG |
| EMB1579-B_F_-RT | AGCAATACGGTTCGCAATC |
| EMB1579-B_R_-RT | GCTTATCGGAATACAAATCGT |
| EMB1579-qRT-F2 | GCGAATTCATGCATAGAGATATGTATTTC |
| EMB1579-qRT-R2 | TAGTCGACCTATAGAGACAATCTAACAAG |
| eIF4A-F1 | CAGAGAACACTCCAACCTGAATC |
| eIF4A-R1 | GGGTATCTATGCTTACGGTTTCG |
| EMB1579-qRT-F1 | TTGAGTTCTCGCCATCCA |
| EMB1579-qRT-R1 | TGGTGAATCAGGTAAAGTAGGG |
| eIF4A-F2 | TGACCAGAGGCTGAATGAAGT |
| eIF4A-R2 | CGTAAGCATAGATACCCCTAAGAA |
| FLC intron1 SF | ATTAGGGCACAAAGCCCTCT |
| FLC intron1 SR | AAGGATCTTGACCAGGTTATCG |
| FLC intron1 UF | AGTTTCCAGTGGCCTTTTCA |
| FLC intron1 UR | AGCATGCTGTTTCCCATATC |
| CYCD2;1-SF | TGGATTCTAAAGGTGGAAGATC |
| CYCD2;1-SR | GAAACAGACACTGCAGCAGCTG |
| CYCD2;1-UF | GATGTTGGTGAGAGAGATTGAG |
| CYCD2;1-UR | GAACCGATCCAAGTAGTTCATG |
| ICK2-SF | GTTCTATGAAGTATAACTTCGATTTCG |
| ICK2-SR | CCTCAAGGAAGTACAAACAAAAG |
| ICK2-UF | GTTGTTGGAATGTTCTATGAAGTAAGTT |
| ICK2-UR | GTGGCTCATCTTTCTCGAAATC |

**Unique restriction enzyme sites are indicated in bold and homologous arm sequences are in italics**
