# Supplementary material for: Phase separation of Arabidopsis EMB1579 controls transcription, mRNA splicing, and development
Source: PLoS Biol. 2020 Jul 21;18(7):e3000782. doi: 10.1371/journal.pbio.3000782 (PMC7413564; doi:10.1371/journal.pbio.3000782)
Supplement: S5 Table — qRT-PCR, quantitative reverse transcription PCR. (DOC) [file pbio.3000782.s020.doc]

**S5 Table. qRT-PCR validation p**rimers

| **Primer Name** | **Primer Sequence** |
| --- | --- |
| CRA1-qRT-F | AGCCCAAATCCAGATCGTAAAC |
| CRA1-qRT-R | TCACCACCGAGAAACCTTGTG |
| CRU3-qRT-F | TAGATGTTCTCCAAGCCACCG |
| CRU3-qRT-R | AACGGAAACACCAACACATCG |
| 2S2-qRT-F | ATTTGCAAGATCCAGCAAGTTG |
| 2S2-qRT-R | AATACATTTAGCCTCAAACATC |
| CesA7-qRT-F | CAGGCGTACTCACAAATGCT |
| CesA7-qRT-R | TGTCAATGCCATCAAACCTT |
| PELPK1-qRT-F | AAAGGTACCGGAGATTCAG |
| PELPK1-qRT-R | CTCAGGCTTTGGAATCTC |
| WRKY48-qRT-F | TCAACATCACCAGCCCTACA |
| WRKY48-qRT-R | CATATCATAACCAAAGCCGGG |
| RAP2.11-qRT-F | TTTCCAATTTACTTTTCCTGATCAATTC |
| RAP2.11-qRT-R | ATCAAGCTTTTGTTGTACGAAACCTTCATTATC |
| MYB36-qRT-F | GGTCCATAATTGCAGCTCAG |
| MYB36-qRT-R | AATCGGTTATGGAGTCTTGACG |
| MYB93-qRT-F | TCTTCCACACAACAATCCGG |
| MYB93-qRT-R | GTCGAAGCAAATGTCAGGCC |
| TPJJ-qRT-F | TTCGGTCGGTGGTAAAGAAC |
| TPJJ-qRT-R | CTTTGGGGAACTTGGAGACA |
| ICL-qRT-F | TGAACACCTCTTCTTCGCTCAGCA |
| ICL-qRT-R | AAAGCTTGCAGAGTTTGACGGTGG |
| CYCB2;2-qRT-F | AGAGGTTCCTCAAGGCAGCTCAAT |
| CYCB2;2-qRT-R | GTGCTGTTCCATTCACTGAAGCCA |
| GLN1;4-qRT-F | GGAGTTGGAGCAGACAAAGC |
| GLN1;4-qRT-R | GATTCCGGCGTAAAGACAAG |
| PAL4-qRT-F | ATCCCGTGACCAACCATGTC |
| PAL4-qRT-R | TGGCAAAGCGCGACTAAGTA |
| MAP18-qRT-F | AAGCCAGCTGTGGAAGA |
| MAP18-qRT-R | TTCGGGAGCCTTAGT |
| PDC1-qRT-F | CAATTGCTGGACTGCAAAGGTG |
| PDC1-qRT-R | AGCAACTCTTTGCTCGTATCATCC |
