# Supplementary material for: Phase separation of Arabidopsis EMB1579 controls transcription, mRNA splicing, and development
Source: PLoS Biol. 2020 Jul 21;18(7):e3000782. doi: 10.1371/journal.pbio.3000782 (PMC7413564; doi:10.1371/journal.pbio.3000782)
Supplement: Attachment — Submitted filename: Reponse to review comments.doc [file pbio.3000782.s023.doc]

**Response to Reviewers’ Comments**

We thank the three anonymous reviewers for their constructive comments. We have taken those comments seriously and tried our best to address the raised concerns and criticisms carefully. We think that the quality of this revised manuscript has been improved substantially. Below, we itemize our responses to the questions.

We thank reviewer #1 for his/her constructive comments.

Reviewer #1:

*The manuscript entitled, “Phase Separation of Arabidopsis EMB1579 Controls Transcription, mRNA Splicing and Development” shows the EMB1579 protein affecting global gene transcription and mRNA splicing. It is quite clear about the phase separation of RMB1579 and its dynamics both in vitro and in vivo. However, there are quite a lot of overstatements on the cellular functions of EMB1579. Additionally, the lack of direct evidence on the EMB1579-MSI4 interaction impact to FLC epigenetic state is another big concern.*

We have tried our best to address the raised concerns; see our detailed responses below.

1)	*It’s not right to claim that phase separation of EMB1579 condenses PRC2, because what they showed are MSI4 and DDB1A, which are actually not PRC2 subunits, only been shown interacting with PRC2 complex.*

Thanks for pointing this out. Indeed, although MSI4 coimmunoprecipitates with CLF (Pazhouhandeh et al., *PNAS*. 2011;108(8):3430-5), it remains uncertain whether MSI4 is part of CLF-PRC2. We changed the statement throughout this revised manuscript.

2)	*It is obvious that EMB1579 functions in flower transition, however, the claim on regulating the H3K27me3 level on FLC through maintaining the PRC2 complex phase transition is a great jump. To claim this, the author should show 1) EMB1579 direct interaction with PRC2 complex proteins, 2) in vivo evidence that the binding of PRC2 subunit proteins on FLC was compromised in emb1579 mutant.*

It was previously shown that MSI4 regulates the flowering time by interacting with CUL4-DDB1 and a CLF-PRC2 complex, and consequently facilitates the role of CLF-PRC2 in establishing and/or maintaining the level of H3K27me3 on the *FLC* locus to repress the expression of *FLC* (Pazhouhandeh et al., *PNAS*. 2011;108(8):3430-5). Several lines of evidence obtained in our study suggest that EMB1579 is involved in the regulation of *FLC*-mediated repression of flowering by physically interacting with MSI4 and recruiting and condensing it through the formation of liquid-like droplets. As MSI4 functions by interacting with CUL4-DDB1 and the CLF-FIE-containing PRC2 complex, we examined the interaction of EMB1579 with CUL4 and DDB1, and CLF and FIE. We found that EMB1579 physically interacts with CUL4 and DDB1, but not the core subunits of CLF-PRC2, CLF and FIE. This finding allows us to propose that EMB1579 functions in *FLC*-mediated repression of flowering by interacting with CUL4-DDB1-MSI4 but not with the core subunits of CLF-containing PRC2. We further demonstrate that EMB1579 droplets can recruit and condense MSI4 and DDB1B. This allows us to speculate that EMB1579 droplets recruit and condense CUL4-DDB1-MSI4 to facilitate their interaction with CLF-PRC2, which in turn promotes the role of CLF-PRC2 in establishing and/or maintaining the level of H3K27me3 on *FLC* to repress the expression of *FLC* and promote flowering.

Certainly, we agree with this reviewer that it is important to determine whether loss of function of *EMB1579* affects the direct binding of CLF-PRC2 to *FLC* chromatin. We are on the way to obtaining the transgenic materials required for this experiment. Specifically, we are trying to introduce the GFP-CLF into *emb1579* mutants by crossing *Arabidopsis* seeds harboring CLF-GFP with *emb1579* mutants and transforming an *EMB1579* CRISPR/Cas9 plasmid into *Arabidopsis* seeds harboring CLF-GFP. Unfortunately, our lab has been closed since January 2020 because of the outbreak of coronavirus in China, and therefore this experiment has been interrupted. We will continue this work after the lab gets restarted. In addition, we realized that measurement of the binding of CLF to *FLC* chromatin will only provide correlative data regarding whether loss of function of EMB1579 impacts the function of CLF-PRC2, as we showed that EMB1579 does not physically interact with the core subunits of PRC2, CLF and FIE. Currently, the functional association of EMB1579 with MSI4 in regulating the level of H3K27me3 on *FLC* chromatin is clear, as we demonstrated that they physically interact with each other and *emb1579* phenocopies *msi4* in terms of the level of H3K27me3, the expression of *FLC* and the flowering time. As it was demonstrated that MSI4 regulates flowering by interacting with CUL4-DDB1 and CLF-PRC2 (Pazhouhandeh et al., *PNAS*. 2011; 108(8): 3430-5), it is reasonable for us to propose that EMB1579 regulates the level of H3K27me3 on *FLC* chromatin by regulating the function of MSI4, which in turn impacts the activity of PRC2, as PRC2 is the only complex responsible for establishing and/or maintaining the level of H3K27me3 on chromatin. We also discussed this issue with an expert on PRC2, Dr. Yue Zhou at the School of Life Sciences, Peking University, who is a former post-doc of Prof. Franziska Turck at the Max Planck Institute for Plant Breeding Research, Department of Plant Developmental Biology, Köln, Germany. Dr. Zhou agrees with our opinion, based on the current data, that it is fair to propose that EMB1579 impacts the function of CLF-PRC2 through regulating MSI4. Furthermore, as this field advances very quickly, and we know that someone has been working on the same protein from the same angle, we are reluctant to wait any longer for this data. Certainly, we realize that many details still need to be revealed regarding the functional relationship between EMB1579 and Polycomb group proteins. We are going to deepen such studies in our lab in the future.

3)	*It is very interesting that the capability of EMB1579△RED to form droplets were compromised, but the EMB1579△RED droplets are even more dynamic than EMB1579 droplets in vitro. How to explain this interesting phenomenon?*

At the moment, we do not actually know why EMB1579△RED droplets are even more dynamic than EMB1579 droplets. But we speculate that it might be due to the alteration in the electrostatic interaction between EMB1579 molecules, as charged residues are enriched in the RED repeat. We discussed this briefly in the Results section.

4)	*Although EMB1579 itself could form phase separation, it does not necessarily mean the droplets observed is initiated by EMB1579, it could be several proteins with the property of forming phase separation co-condense, and the RED domain responsible for the multi-valent interaction. Thus, EMB1579△RED lost the ability of forming phase separation in vivo could due to the lack of the interaction with these proteins, and the biological function defects in EMB1579△RED may also be explained by the RED domain as the interacting domain with other transcription/splicing complex proteins. Thus, it is not right to claim that “we uncover a direct link between the LLPS property and their physiological functions”.*

We agree with this reviewer that we cannot completely rule out the possibility that deletion of the RED domain might impair the interaction of EMB1579 with other proteins that consequently impairs the multi-valent interaction between EMB1579 and the LLPS of EMB1579. But based on the newly generated data showing that EMB1579ΔRED retains the ability to interact with the functionally relevant interactors (see the newly prepared S7 Fig and S8C Fig), the functional deficiency in EMB1579ΔRED is likely due to the fact that it cannot form normal liquid-like droplets. Certainly, we agree with the reviewer that it might be inappropriate to claim that “we uncover a direct link between the LLPS property and their physiological functions”, so we tried to soften our statement in this revised manuscript.

5)	*The authors should specify how they justify “both transcription and mRNA splicing defects in emb1579 mutants suppress the later flowering phenotype in emb1579 mutants”, because only expression level of FLC was shown in fig S4.*

We found that both transcription (Fig. 2C) and mRNA splicing (Fig. 2E, 2F) of *FLC* are altered in *emb1579* mutants. Our finding is actually similar to previous observations that loss of function of RNA binding proteins *RZ-1B* and *RZ-1C* increases the expression of *FLC* and causes defects in pre-mRNA splicing of *FLC* (Wu et al., Plant Cell. 2016; 28(1): 55-73). We thus speculate that the alterations in the transcription and pre-mRNA splicing of *FLC* greatly contribute to the later flowering phenotype in *emb1579* mutants. In support of this speculation, we found that reducing the level of *FLC* transcripts using RNAi rescues the late flowering phenotype in *emb1579* mutants to a large extent (S4 Fig). We realized that reducing the amount of *FLC* transcripts using RNAi actually eliminated both the transcriptional and pre-mRNA splicing defects of *FLC*. At the moment, we do not know how to specifically eliminate the pre-mRNA splicing defects of *FLC* in *emb1579* mutants to see whether and to what extent it contributes to the late flowering phenotype in these mutants.

Minor points:

1)	*It is very interesting that the homolog of EMB1579, MAP190 could interacts with actin filaments and microtubules. EMB1579 could form phase separation in vivo, the actin and microtubules are also shown to form phase separation1,2, this could be a very interesting area to further link the phase separation of nucleus skeleton with transcription/splicing.*

*1	Weirich, K. L. et al. Liquid behavior of cross-linked actin bundles. Proceedings of the National Academy of Sciences 114, 2131, doi:10.1073/pnas.1616133114 (2017).*

*2	Guharoy, M., Szabo, B., Martos, S. C., Kosol, S. & Tompa, P. Intrinsic Structural Disorder in Cytoskeletal Proteins. Cytoskeleton 70, 550-571, doi:10.1002/cm.21118 (2013).*

We agree with this reviewer that it will be interesting to examine the role of phase separation of the nucleus skeleton in regulating transcription/splicing via careful and deep analysis of EMB1579. Related studies are on the way in our lab. We discussed this point briefly in the Discussion section and we cited those references.

We thank reviewer #2 for his/her constructive comments.

Reviewer #2:

*In this manuscript, Zhang et al. delve into functions of a plant-specific protein, EMB1579. They find that EMB1579 deletion mutants exhibit global changes in transcription and mRNA splicing. These phenotypes correlate with organismal defects in seedling growth and flowering. The authors find that EMB1579 forms punctate structures in cells that exhibit some liquid-like behaviors, such as high internal mobility and fusion. Cellular EMB1579 compartments co-localize with transcription and splicing factors, including PRC2 complex proteins MSI4 and DDB1B. These proteins also appear to directly interact in Arabidopsis. The authors show that EMB1579 protein phase separates to form distinct condensates in vitro. The in vitro EMB1579 condensates also concentrate MSI4 and DDB1B, suggesting that the cellular and in vitro compartments have certain similarities. The authors further identify a region in EMB1579, RED repeats, which alters aspects of EMB1579 phase separation in cells and in vitro. RED repeat deletion mutant does not fully rescue the defects of EMB1579 deletion mutant, indicating that this region is indeed important for the function of EMB1579. In summary, this study describes a novel function of EMB1579 in transcription and mRNA splicing, which correspond to interesting plant phenotypes. In addition, the fact that the cellular tendency of this protein to localize to nuclear foci correlates with its ability to phase separate in vitro is interesting. However, as I outlined in detail below, many of the conclusions around the functional significance of EMB1579 phase separation or the link between EMB1579’s molecular function and plant phenotypes are not strongly supported. Moreover, the intro and discussion are currently plant-specific and could be expanded to be accessible to a broader PLOS Biology audience. Apart from these general comments, several points stood out to this reviewer as critical to address.*

Thanks for pointing this out. We agree with this reviewer that it is important to expand our finding to a broader PLOS Biology audience. Specifically, we mentioned briefly how our findings might enhance our understanding of the function of LLPS in regulating transcription and mRNA splicing in the Introduction section. In addition, we also tried to link our findings to previous studies on PRC2 complex in both the Introduction and Discussion sections.

Major comments:

•	*The intro and discussion will benefit from linking the findings from the current study to what is already known out there. Please consider expanding on the general implications of the findings.*

That’s a great suggestion. We tried to put our study in the context of regulation of transcription and pre-mRNA splicing mediated by LLPS mechanisms. Specifically, we also tried to link our study to the regulation of the PRC2 complex, which has been shown to be important for establishing the gene transcription patterns of developmental programs in different organisms.

o	*PCG complex proteins have been shown to phase separate before, which is not at all mentioned in the manuscript.*

Thank you for the suggestion. We cited the references and discussed briefly the potential coordination of LLPS of EMB1579 with the LLPS system of PCG complex proteins.

o	*what are some analogous nuclear bodies or proteins in non-plants and what do we know about them (such as other PRC proteins that phase separate); what other analogous RED repeat containing proteins exist and what do they do; specify which proteins or nuclear compartments have been shown to use this mechanism to phase separate…etc.*

We had actually already mentioned the function of some RED repeat-containing proteins in the Discussion section. As we mentioned in the Discussion section, those proteins were shown to form compartments in the nucleus, but it remains to be characterized whether those proteins form compartments using the LLPS mechanism. To date, related studies are rather limited in the literature. In this regard, our study sheds light on the potential mechanism of action of those proteins in a different physiological setting.

•	*This study doesn’t specifically test the role of IDRs in phase separation, so please downplay the link between IDRs and phase separation in the text (currently it implies that the high degree of disorder in EMB1579 may contribute to its phase separation)*

We agree with this reviewer that we did not investigate the effects of IDRs on the LLPS of EMB1579, although some deep characterization is on the way in the lab right now. We tried to tone down our statement in this revised manuscript.

•	*The conclusions rely on in part findings in Figure 4, in which the authors look at changes in transcription and splicing in ‘phase separating conditions’. To support the conclusions, it will be crucial to show that EMB1579 phase separation in plant extracts is not disrupted during pull-down and that the assay isolates specifically the phase separated compartments. Without this information we can’t conclude that these interactions specifically occur in phase separated compartments. Consequently, I also do not agree that the model in Fig 4 is well supported.*

We agree with this reviewer that it is important to demonstrate whether the phase separation of EMB1579 is not disrupted during the incubation with the total extract. We do not have direct data to address this point. However, we do have data to show that EMB1579 droplets formed under the same conditions as they formed *in vitro* can condense RFP-MSI4 in *Arabidopsis* total extract (Fig. 6H). To some extent, this supports the idea that some of the pulled down EMB1579 is in the phase separated condition. In addition, we tried to soften the statement by using the term “pulled down fraction” in this revised manuscript. We agree that more experiments need to be done to firmly support this model. But we have data to show that some pulled down proteins physically interact with EMB1579 and colocalize with EMB1579 droplets *in vivo*. Taking MSI4 as an example, we demonstrate that it functions in the same pathway with EMB1579, and we demonstrate that EMB1579 is required for the formation of MSI4 compartments in *Arabidopsis* protoplasts. Based on those data, we believe that it is reasonable to make a simple model in which EMB1579 regulates the function of those interactors by forming compartments.

•	*Some of the key findings rely on the differences between WT and RED deletion mutant. However, I could not find information on whether the expression and localization of these proteins are comparable in plants. It is essential to show this with quantification in Fig 4, does the RED mutant express at the same level? From the staining, it looks like expression of the mutant may be lower. Please show by western blot and quantification.*

As suggested, we performed the western blot analysis with an anti-GFP antibody, and we found that the amount of EMB1579-GFP and EMB1579ΔRED-GFP protein is roughly similar, as shown in the newly prepared Fig. S6B.

•	*Relating to the in vitro phase separation assays, it would be important to include whether phase separation occurs at near physiological protein and salt concentrations. i.e. measure approximate concentration of the protein in plant cells.*

As suggested, we measured the concentration of EMB1579 in the nucleus and found that it was approximately 33 nM (see the newly prepared data in S5 Fig). This suggests that EMB1579 can undergo phase separation on its own, as the critical concentration for the LLPS of EMB1579 can go down to 25 nM or less.

•	*In Fig 2K, there are number of in vitro EMB1579 condensates that are touching but not fusing in both the still images and in the montage. The shapes of the condensates are generally not spherical either. This is not what you expect from dynamic liquid droplets and contradictory to the rapid fusion seen in cells, please comment on this and show additional quantification to support sphericity or fusion dynamics.*

We re-performed the LLPS of EMB1579 *in vitro*, and found that we can detect fusion events between EMB1579 droplets and the droplets are spherical. We replaced the original results with the new data in Fig 2K and S4 Movie.

•	*The fact that the RED mutant does phase separate to form puncta in vitro but not in cells was strange to this reviewer. Also the mutant condensates do not grow in size in vitro as concentration increases - this is surprising given the principles of phase separation and suggest that RED repeats may have specific roles in condensate growth, rather than LLPS. Please comment on this and adjust the model accordingly.*

We found that EMB1579 without the RED repeat is still able to form puncta, but the puncta are small, as shown in Fig. 5H-J. We do not know exactly how the RED repeat might contribute to the LLPS of EMB1579. It might modulate the interactions between individual EMB1579 molecules through electrostatic interaction, as charged residues are enriched in the RED repeat. We discussed this briefly in this revised manuscript. We realized that this protein is very interesting as the IDRs occupy most of the protein and the formed droplets are extremely dynamic. We are going to put more effort into revealing how the protein sequences of EMB1579 contribute to its LLPS and the formation of functionally competent liquid-like droplets. Specifically, we want to determine how the RED repeat is involved in the nucleation, growth and maturation of EMB1579 droplets. This might help us to develop a more precise model regarding the dynamic formation of EMB1579 droplets.

*Related to this, the following statement is not supported by the data: "These data together suggest that the RED repeat is required for the LLPS property of EMB1579 in vitro”*

We changed this statement to “These data suggest that the RED repeat is required for EMB1579 to form normal liquid-like droplets”.

•	*In several places the authors make the conclusion that the LLPS of EMB1579 is essential for its function: e.g. "These data together suggest that the cellular functions of EMB1579 depend on the formation of appropriate liquid-like compartments.” The authors showed that RED repeats alter EMB1579 phase separation in vitro and EMB1579 mutant plants have defects that can be partly rescued by the mutant. With current data, it could very well be that the RED repeats are important for interaction with RNA or other proteins that are essential for EMB1579 function. Pull-down experiments to show that EMB1579 RNA and protein interactions are unaffected could be additional support for their conclusion. However, ultimately, authors would need to show that these reactions are occurring ‘better’ in the condensate in vitro or in cells in order to make this conclusion. Otherwise, please soften the conclusion.*

Thanks for this suggestion. We tried to address this concern in two ways. First, we tried to soften our conclusion throughout the manuscript. Second, we used the luciferase assay to examine the interaction of 20 functionally relevant EMB1579 interactors with EMB1579ΔRED. We found that the interactions were not disrupted, as shown in the newly prepared S7 Fig. Considering this result along with our finding that deletion of the RED repeat does not disrupt the interaction between EMB1579 and MSI4 (see the data in S8C Fig), we can speculate that the functional failure of EMB1579ΔRED is due to its inability to form normal droplets rather than its inability to interact with functionally relevant partners.

•	*This reviewer did not find the trimethylation defects very convincing in support of the following conclusion: "These data together allow us to propose that EMB1579 droplets condense PRC2 to facilitate its activity in maintaining the correct level of H3K27 trimethylation on FLC to regulate the transcription of FLC and flowering (Fig 6H).” Please measure trimethylation defects with multiple methods and quantify.*

We measured the level of H3K27me3 by western blot probed with an anti H3K27me3 antibody and found that the global level of H3K27me3 on chromatin is reduced in *emb1579* mutants compared to WT (see the newly prepared results in Fig. 6D). This further supports our conclusion that EMB1579 is involved in flowering via maintaining the level of H3K27me3 on the *FLC* locus.

•	*The conclusion that EMB1579 condensates function to “promoting PRC2 activity in maintaining the level of H3K27 trimethylation on FLC” is too strong – the current study shows correlative data only.*

Yes, we only have the correlative data to support the role of EMB1579 in regulating the level of H3K27me3 on *FLC* mediated by PRC2. As suggested, we softened the conclusion in this revised manuscript.

Minor comments:

•	*A number of grammatical errors are found throughout the manuscript – please comb through and correct*

A native English speaker has helped to copyedit the revised manuscript and the grammatical errors have been corrected.

•	*Figures heavily rely on the legends to make sense – more detailed labelling within the figures would significantly enhance the readability. e.g.) add arrows, label which proteins are represented in the microscope images, are gels representing WB or qPCR etc.*

We have added more labels to the figures in this revised manuscript.

o	*Fig 1B: "distorted cell division and cell expansion" It’s difficult to see what cell division and expansion defects should be seen in the images - is it increase or decrease in cell division or both? Please include arrows.*

We added white triangles to indicate the defects in cell division and cell expansion. In particular, we quantified the number of meristematic cells in root (see the newly generated data in Fig. 1G), which suggests that cell division is impaired in *emb1579* mutants.

o	*Fig 1C: Not sure what kinds of seed defects are found. Please add arrows*

We found that more wrinkled seeds appear in *emb1579* mutants. These are indicated by white triangles in the revised figure.

•	*Conclusions in the text are written with very vague language, making it difficult to understand what the authors are trying to convey. Please be more precise. e.g.) "distorted cell division and cell expansion" - is it increase or decrease or both?; "dynamic changes during the cell cycle” – what is dynamically changing? etc.*

Thanks for pointing this out. We have tried to make clearer statements in this revised manuscript.

o	*Fig 2C: Please specify what changes are referred to by "dynamic changes during the cell cycle”. The only difference that is obvious to this reviewer is that GFP intensity decreases sometimes, which doesn’t come back – this doesn’t seem very ‘dynamic’.*

In the revised manuscript, we have clearly explained the changes in the intracellular localization pattern of EMB1579 during the cell cycle. Specifically, EMB1579 disappears as the nuclear membrane breaks down, and re-concentrates in the nucleus as the nuclear membrane re-forms.

•	*Fig 2D: Image does not match what is reflected in quantification in Fig 2E - I see larger bodies in root meristem zone. In 2E, is the difference significant?*

Thanks for pointing this out. Indeed, we found that the original images shown in Fig 2D are not typical; we therefore replaced them with new images in the revised Fig. 2.

•	*Fig 2J: The purified proteins have multiple ‘contaminating bands’ - please comment on this.*

The contaminating bands might be due to protein degradation. As suggested, we commented on this in the legend of Fig 2J in this revised manuscript.

•	*In text corresponding to Fig 5G, the authors conclude that "EMB1579ΔRED droplets are even more dynamic than EMB1579 droplets”. The RED mutant also has less mobile fraction. Please comment on this.*

We discussed this briefly in this revised manuscript.

•	*In discussion, “it might be fair for us to speculate that the extraordinarily dynamic EMB1579 droplets enable sessile plants to rapidly alter their DNA- and RNA-based activities to control transcription and pre-mRNA splicing in response to external and internal stimuli.” This is not fitting their conclusion that RED deletion mutant was more dynamic – ie. there’s no functional data showing link between changes in dynamics and transcription/pre-mRNA splicing control*

We meant to compare EMB1579 droplets to their counterparts with similar functions in other organisms, and found that EMB1579 droplets turnover much rapid than them, such as droplets formed by nucleophosmin (NPM1) and the RNA binding protein hnRNPA1, which motivates us to speculate that it might enable EMB1579 to function within the context of plant cells. Indeed, deletion of the RED repeat increases the dynamics of EMB1579 droplets, but their size is reduced substantially and they do not truly exist in cells. This on the other hand suggests that the dynamics and size of liquid-like droplets need to be maintained at certain extent to enable them to function properly in cells.

We thank reviewer #3 for his/her constructive comments.

Reviewer #3:

The experiments in this manuscript are appropriately designed and carried out, and the findings are significant. This work will be of great interest to the general readers of PLOS Biology as well as those interested in the biochemistry/biophysics of LLPS and it physiological role. I will recommend it for publication.

Response: Thanks for the encouraging comments.

Comments:

*I would like to suggest to the authors to added a figure or two showing a phase separation prediction for EMB1579 protein. A recent review from my former colleagues summarize the available predictors:*

*First-generation predictors of biological protein phase separation by Vernon and Forman-Kay. Curr Opin Struct Biol (2019)*

*Considerations and challenges in studying Liquid-Liquid Phase Separation and Biomolecular Condensates by Alberti, Gladfelter and Mittag. Cell (2019) doi: 10.1016/j.cell.2018.12.035.*

We reanalyzed the protein sequences according to the phase separation predictors mentioned in those papers. See the newly prepared data in Fig. 2I. We also cited the related papers.

Minor:

*there are no page numbers in the manuscript some image quality is fuzzy especially if you magnify (e.g. fig 3A)*

Thanks for pointing this out. We added page numbers in this revised manuscript. We uploaded high resolution images, but the resolution of the images is reduced during conversion into the PDF file by the *PLOS Biology* editorial system.

*PCR2 was not defined when first encounted in the text. It was done later.*

We fixed this problem in the revised manuscript.

*Because nucleic acids can also undergo LLPS, the following statement should be changed to reflect that fact:*

*“The biophysical mechanism underlying the formation of non-membranous subnuclear compartments is liquid-liquid phase separation (LLPS), which is mediated by collective protein–protein and protein–nucleic acid interactions as well as nucleic acid-acid interactions[15, 18]”. One relevant reference is “ RNA phase transitions in repeat expansion disorders by Jain and Vale. Nature (2017) doi: 10.1038/nature22386*

Thanks for pointing this out, we included this reference in the revised manuscript.
