# Supplementary material for: Phase separation of Arabidopsis EMB1579 controls transcription, mRNA splicing, and development
Source: PLoS Biol. 2020 Jul 21;18(7):e3000782. doi: 10.1371/journal.pbio.3000782 (PMC7413564; doi:10.1371/journal.pbio.3000782)
Supplement: Attachment — Submitted filename: Response to Reviewers comments.docx [file pbio.3000782.s024.docx]

**Response to Reviewer’s Comments**

We thank Reviewer 2 for his/her comments.

*Reviewer #2: The manuscript presents beautiful cell biological and developmental characterization of EMB1579 'KO' plant mutants. These mutants show changes to global transcription and RNA splicing. The authors also identify a number of proteins that interact with EMB1579, including DDB1 and MSI4. The finding that it does not interact with some of the PRC2 complex proteins also shed more light into its functional mechanism. It is striking that RED mutant EMB1579 do not rescue the defects of EMB1579 KO plants. These findings are valuable and form a strong foundation for future studies. The result that EMB1579 phase separates into very dynamic condensates is convincing, and the requirement of RED domains to make large droplets is interesting, particularly in light of their inability to rescue KO plants. The fact that these condensates can also partition DDB1 and MSI4 recapitulates in vivo findings, and thus valuable. However, the presented data does not demonstrate that the EMB1579 phase separation facilitates EMB1579 functions or how.*

Thanks for the encouraging comments. We have tried our best to address the concerns you raised, see below.

*This revised manuscript has meaningful additions from the last version. In my view, the focus on EMB1579 phase separation takes away from the novelty of findings mentioned above. Importantly, the manuscript still has a number of overstatements linking EMB1579 phase separation to its function, which is a big concern. Moreover, the manuscript would greatly benefit from a significant restructuring to streamline presentation. Please find detailed comments below.*

Thanks for pointing this out. We agree with this reviewer that the focus on phase separation of EMB1579 takes away from the novelty of findings presented in the manuscript. We thus reorganized the writing in the INTRODUCTION section, to improve the readability and logical flow of this story. In addition, we tried to fix the overstatements throughout the manuscript. For details, please see the revised manuscript and the point-by-point response below.

*A few examples of overstatements - many appear in titles. Please comb through the manuscript for additional mistakes.*

1.	*"Many nuclear proteins bind to EMB1579 droplets and loss of function of EMB1579 affects global transcription and mRNA splicing in Arabidopsis" ◊ There is no validation that the method isolates interactions that occur in EMB1579 condensates. There is usually a considerable amount of protein still in the dilute phase in the in vitro assays. As such, the authors would need to show that they concentrated EMB1579 condensed phase before identifying interactors to make this claim. Relating to this, please report the partition coefficient.*

We incubated the preformed EMB1579 droplets with the total *Arabidopsis* root extract under the conditions that induce phase separation of EMB1579 *in vitro*. We did not check whether and to what extent the EMB1579 droplets underwent change during the incubation. However, we do have data to show that EMB1579 droplets, under the same conditions which induce droplet formation *in vitro*, can condense RFP-MSI4 in *Arabidopsis* total extract (Fig. 6H). This supports the idea that some of the pulled down EMB1579 is in the phase separated condition. In addition, we performed the *in vitro* recruitment and condensation experiments of EMB1579 condensates using DDB1 and MSI4 as the substrates and calculated the partition coefficient. We found that the value of the partition coefficient is very high for EMB1579 and the recruited DDB1 and MSI4 (Fig. 6J and S9B Fig), which suggests that most of the proteins will likely enter EMB1579 condensates during the incubation. Certainly, we cannot guarantee that all cosedimented proteins enter the EMB1579 droplets, but the level of partitioning was sufficient for us to perform subsequent validation. Furthermore, we tried to soften the statement by using the term “pulled down fraction” in the manuscript.

2.	*"formation of normal-sized EMB1579 droplets is crucial for its cellular functions"; "The formation of EMB1579 droplets is crucial for its cellular functions" ◊ The findings demonstrate that the RED domain is crucial for rescuing EMB1579 KO phenotypes, and this correlates with the RED mutant's inability to form large EMB1579 condensates. To make this claim, the authors would need to show that RED mutant is equally 'functional' as full length, only disrupted in its ability to condense, but still cannot rescue EMB1579 KO phenotypes.*

We actually demonstrate that EMB1579ΔRED is still able to interact with the functionally relevant interactors (see the data in S7 Fig). These data allow us to conclude that the functional deficiency in EMB1579ΔRED is likely due to its inability to form droplets.

3.	*"we demonstrate that the formation of normal EMB1579 droplets is crucial for the cellular functions of EMB1579 (Fig 5K; S6C-6F Fig)" ◊ RED domain mutant was introduced in EMB1579 KO cells to draw this conclusion. It is equally likely that RED mutant has defects in mediating EMB1579 functions.*

We actually cannot completely rule out the possibility that EMB1579ΔRED has defects in mediating EMB1579 functions. However, based on the data that EMB1579ΔRED retains the ability to interact with the functionally relevant interactors (see the statement above), we believe that it is fair to argue that the formation of normal sized EMB1579 droplets is crucial for the cellular functions of EMB1579.

*4.	"EMB1579 compartments condense different biomolecules to regulate their functions" ◊ The authors only showed that different proteins co-localize; regulation was not shown.*

We initially demonstrated that some proteins pulled down with EMB1579 condensates colocalize with EMB1579 compartments (Fig 4A). Subsequently, we demonstrated that some of these colocalized proteins can be recruited and condensed into EMB1579 condensates *in vitro* (Fig 6G and S9B Fig). Importantly, using MSI4 as the representative protein, we demonstrated that loss of function of EMB1579 inhibits the formation of MSI4 compartments *in vivo* (Fig 6I), which suggests that EMB1579 is required for these proteins to form compartments *in vivo*. Using floral transition as the physiological process, we demonstrated that loss of function of *EMB1579* phenocopies *msi4* in terms of flowering time, the level of H3K27me3 on the *FLC* locus and transcription of *FLC*. These data together suggest that EMB1579 regulates the function of those proteins via formation of condensates *in vivo*.

*5.	Please consider replacing 'droplets' with 'condensates' or the like, which are more specific and accepted term.*

We replaced 'droplets' with 'condensates' in this revised manuscript as suggested.

*The introduction did not provide sufficient background information relevant to the study at hand and was too general and vague at places. Please implement a clear flow, clearly stating the knowns and unknowns to set the stage for the study and relating the subject of interest to other areas of biology (including non-plant).*

The main finding of our story is that LLPS of a nucleus-localized protein controls gene transcription and mRNA splicing, which directly links nuclear subcompartmentalization to gene transcription and mRNA splicing in plants. Certainly, some important players within the gene transcription and mRNA splicing machineries are able to undergo LLPS, such as transcription factors. We cited the relevant references in the INTRODUCTION section. But, our findings show that LLPS of a nucleus-localized protein, which is not directly involved in gene transcription and mRNA splicing, regulates gene transcription and mRNA splicing via condensation of protein components of the gene transcription and mRNA splicing machineries. Our study thus provides a new mechanism for regulating gene transcription and mRNA splicing.

6.	*"the molecular mechanisms underlying the organization of the compartments, what functions they can perform, and how they perform their functions remain to be documented." ◊ This is inaccurate. Numerous molecular mechanisms underlying condensate formation has been reported in the last decade and many functions have been demonstrated, and how they do this. This information for individual condensates may not be well understood.*

We fixed this problem in this revised manuscript.

7.	*Transcription and RNA splicing have been linked to LLPS, which seems very relevant given that EMB1579 seems to control these processes. These studies are not at all mentioned or discussed.*

We cited several references about the link between LLPS and gene transcription and mRNA splicing in the INTRODUCTION section. Due to the space limitation, we did not expand this aspect of the writing. This is different from our findings that LLPS of a nucleus-localized protein condenses protein components of the transcription and mRNA splicing machineries to control transcription and mRNA splicing.

8.	*Cul4-DDB1-MSI4 is a major focus of this study. Cul4-DDB1 is also a well-characterized E3 Ub-ligase, however this is not mentioned. Is this how the complex regulates histone modifications? Is MSI an F-box protein?*

Yes, Cul4-DDB1 is an E3 Ub-ligase that is involved in the regulation of flowering time via association with the CONSTITUTIVELY PHOTOMORPHOGENIC1 (COP1)-SUPPRESSOR OF PHYA (SPA) complex to control CONSTANS protein abundance (Chen et al., The Plant Cell, 2010, 22: 108–123). We mentioned this finding in the revised manuscript. MULTIPLE SUPPRESSOR OF IRA (MSI) is a homologue of nucleosome remodeling factor 55-kDa (p55), which is a core component of *Drosophila* PRC2 complex.

9.	*EMB1579 is introduced abruptly in the intro. The flow of the results section was rough; it jumped from EMB1579 phase separation to cellular functions and back, with obvious missing pieces in linking the two concepts.*

Thanks, we have fixed this problem by introducing the information about EMB1579 first in the INTRODUCTION section.

10.	*"embryonic developmental defects at different stages, resulting from distorted cell division and cell expansion in emb1579 embryos compared to WT (Fig 1B). Specifically, we found that the division plane of cells was mispositioned and cells were swollen in emb1579 mutants (Fig 1B)" ◊ Please point to distorted cell division, expansion, division plane, swelling*.

We have added more symbols to indicate the cellular defects in the revised figure.

11.	*"We initially analyzed the amino acid sequence of EMB1579 using different phase separation predictors reported previously [47, 48] and found that it contains IDRs that occupy most of the protein (Fig 2I)." ◊ The cited predictors do not look at the simple presence of IDRs. They report the likelihood of a protein to phase separate based on specific parameters. If the predictors were used, please state what was found or cite correct references for protein disorder prediction.*

Thanks for pointing this out, we cited the right references in this revised manuscript.

12.	*The authors state that EMB1579 phase separates at 25nM and exist in cells at around 33 nM. One would predict that EMB1579 is phase separating all the time, how could this regulate specific gene transcription and splicing? Also, the in vitro condensates recover fluorescence much faster than in cells, highlighting that there are other components of the condensates that likely play important roles (at least for the material properties).*

By comparison with data in the literature, EMB1579 condensates are very dynamic, but they still function via recruiting and condensing other important players in cells, which we demonstrated to some extent in this manuscript. For instance, we found that EMB1579 is necessary for MSI4 to form compartments in cells (Fig 6I). However, we still know very little about this protein, and its function is definitely worth exploring in the future. I agree that it is interesting to know whether there are other components within the condensates that play roles in regulating the LLPS dynamics of EMB1579 *in vivo*. We are currently searching for binding partners of EMB1579 that may impact the dynamics of EMB1579 droplets.

13.	*"Taking these data together, we propose that EMB1579 undergoes LLPS to form liquid-like droplets that condense biomolecules crucial for chromosomal function and RNA biology to control nuclear events, such as transcription and pre-mRNA splicing (Fig 4C)." ◊ I would like to see a model like this after all relevant data is presented.*

We have shown that (1) EMB1579 forms compartments both *in vitro* and *in vivo*; (2) many DNA and RNA binding proteins are enriched in the compartments; (3) loss of function of *EMB1579* causes global alterations of gene transcription and pre-mRNA splicing; and (4) formation of normal sized droplets is crucial for the function of EMB1579. Therefore, we believe that it is fair for us to propose a rough model like the one shown in Fig 4C. Subsequently, we aimed to reveal how exactly EMB1579 is involved in the regulation of a specific physiological process, namely floral transition. Specifically, we focused on the role of EMB1579 in *FLC*-mediated repression of flowering, and we demonstrated that EMB1579 droplets are able to condense MSI4, which is involved in the autonomous flowering pathway via regulating the expression of *FLC*. EMB1579 facilitates the role of MSI4 in maintaining the level of H3K27me3 on the *FLC* locus and the transcription of *FLC*. We thus propose another model to illustrate how EMB1579 is involved in the regulation of *FLC*-mediated repression of flowering, as shown in Fig 6J.

Discussion:

14.	*"MSI4 regulates the flowering time by forming CUL4-DDB1MSI4 complex that interacts with a CLF-PRC2 complex to repress the expression of FLC via H3K27 methylation" ◊ Please include speculations as to how this works? Ub-ligase function or not?*

MSI4 is involved in the control of flowering time by regulating the expression of *FLC*. MSI4 interacts with CUL4-DDB1 to form a complex named CUL4-DDB1^MSI4^, which subsequently interacts with a CLF-PRC2 complex to facilitate its role in promoting trimethylation of H3K27 on *FLC*. This work was published by Pazhouhandeh et al., *PNAS*. 2011; 108(8): 3430-5. We described those findings in the INTRODUCTION section.

CUL4 functions in the regulation of flowering via controlling the abundance of CONSTANS protein through its E3 ligase function. *cul4* single mutants exhibits an early flowering phenotype. However, loss of function of *CUL4* enhances the phenotype of *FLC*-mediated repression of flowering through a CLF-PRC2 complex in *msi4* mutants.

15.	*"we speculate that EMB1579 performs its cellular functions by forming droplets that can recruit and condense CUL4-DDB1MSI4 complex to increase their local concentration. This will facilitate the interaction of CUL4-DDB1MSI4 complex with CLF-PRC2 complex to promote its role in establishing and/or maintaining the level of H3K27me3 on FLC, thereby repressing FLC transcription and promoting flowering (Fig 6J)" ◊ The authors showed that EMB1579 does not bind PRC2 components, how would this work?*

It was shown previously that MSI4 functions in regulating floral transition via interacting with CUL4-DDB1 and a CLF-PRC2 complex (Pazhouhandeh et al., *PNAS*. 2011; 108(8): 3430-5). Our study showed that (1) EMB1579 physically interacts with MSI4; (2) EMB1579 condensates can recruit and condense MSI4 *in vitro* and *in vivo*; and (3) *emb1579* phenocopies *msi4* in terms of the flowering time, the level of H3K27me3 on the *FLC* locus and the transcription of *FLC.* Together, these results suggest that EMB1579 regulates the transcription of *FLC* via controlling the function of MSI4, which in turn impacts the function of CLF-PRC2 in establishing and/or maintaining the level of H3K27me3 on the *FLC* locus.
